# Quantitative modelling of P-TEFb mediated CTD phosphorylation identifies local cooperativity

**DOI:** 10.1371/journal.pcbi.1014531

**Published:** 2026-07-30

**Authors:** Aaron Callenbach, Domagoj Dorešić, Robert Düster, Vanessa Nakonecnij, Erika Dudkin, Matthias Geyer, Jan Hasenauer

**Affiliations:** 1 Life and Medical Sciences (LIMES) Institute, University of Bonn, Bonn, Germany; 2 Bonn Center for Mathematical Life Sciences, University of Bonn, Bonn, Germany; 3 Institute of Structural Biology, University of Bonn, Bonn, Germany; Inria Saclay: Inria Centre de Recherche Saclay-Ile-de-France, FRANCE

## Abstract

Fine-tuned regulation of RNA polymerase II (Pol II) activity is essential for accurate gene expression. A key layer of this regulation involves phosphorylation of Pol II’s C-terminal domain (CTD), a repetitive heptapeptide tail that coordinates transcription and RNA-processing factors. The kinase P-TEFb plays a major role in this process, yet its precise phosphorylation mechanism remains unclear. Previous *in vitro* studies have suggested a distributive mode of action based largely on qualitative inspection of mass spectrometry data rather than quantitative analysis. Here, we use mathematical modelling of CTD phosphorylation to explore whether local context, such as neighbouring phosphorylations or directional biases, affects P-TEFb activity on the CTD. Our results indicate that P-TEFb acts distributively but with pronounced local cooperativity: repeats adjacent to phosphorylated sites are modified at higher rates. We find no evidence for directional bias, although the limited positional resolution of the data precludes a definitive conclusion. These results identify local context as an important factor in P-TEFb-mediated CTD phosphorylation and establish a quantitative modelling framework for dissecting multi-site modification dynamics.

## Author summary

Gene expression in human cells is a carefully coordinated process that ensures the right genes are activated at the right time. Central to this process is RNA polymerase II, a multi-protein complex that transcribes DNA into precursors of messenger RNA. Its largest subunit – RPB1 – possesses a flexible tail, called the C-terminal domain (CTD), which acts as a platform for other proteins that help control transcription. To function properly, the CTD must be decorated with chemical marks, most importantly phosphate groups, by enzymes known as kinases. In our study, we focus on one of these kinases, called P-TEFb, which plays a key role during transcriptional elongation. Previous work suggested that P-TEFb adds phosphate groups in a random, distributive fashion, but it was unclear whether its activity depends on the surrounding phosphorylation state. Using mathematical models trained on experimental data, we find that P-TEFb tends to add new phosphate marks next to sites that are already modified. This means that its activity is locally cooperative rather than purely random. Our results suggest that the local chemical environment of the CTD helps guide the formation of phosphorylation patterns during gene transcription.

## Introduction

**Fig 1:**
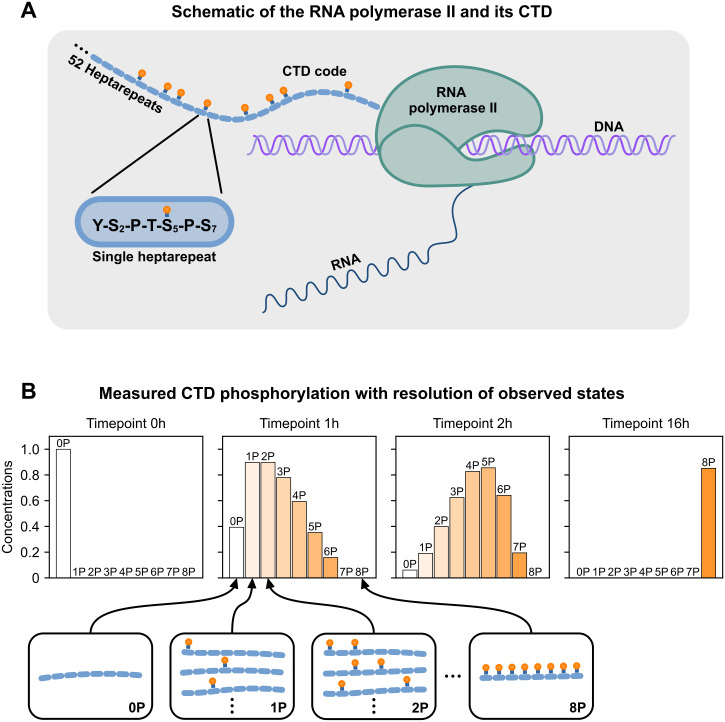
Schematic of the RNA polymerase II CTD and measured phosphorylation dynamics. **(A)** Schematic of the RNA polymerase II and its CTD: Blue dashes denote 52 heptarepeats of the human wild-type CTD. Orange marks represent phosphorylation of those heptarepeats. **(B)** CTD phosphorylation dynamics measured by mass spectrometry: mass spectrometry resolves CTD populations with 0–8 phosphorylations at different time points (0 h, 1 h, 2 h, 16 **h)**. Bar plots show the relative abundance of phosphorylation configurations with 0 to 8 phosphorylations, with schematic CTD representations below (white-to-orange gradient indicates increasing phosphorylation).

Accurate gene expression is fundamental to cellular identity, development, and response to environmental signals. In eukaryotes, transcription by RNA polymerase II (Pol II) is tightly coordinated with co-transcriptional RNA processing. Pol II is a multi-protein complex composed of twelve subunits (RPB1-RPB12) responsible for the transcription of protein-coding genes, as well as many non-coding RNAs [1]. Its largest subunit, RPB1, includes a repetitive tail known as the C-terminal domain (CTD), consisting of tandem heptarepeats of the seven-amino-acid sequence Tyr–Ser2–Pro–Thr–Ser5–Pro–Ser7 (YSPTSPS) (Fig 1A). The CTD undergoes dynamic phosphorylation that coordinates the transcription process [2]. A distinct set of transcriptional Cyclin-dependent kinases (CDKs) directly regulates Pol II by phosphorylating its CTD. These CDKs thereby promote or preclude the binding of proteins to the CTD, enabling the specific recruitment of appropriate transcription factors throughout the transcription process [2].

During transcription initiation, Ser5 residues of the CTD are phosphorylated by the Kin28/CDK7 subunit of transcription factor IIH and by the Srb10/CDK8 subunit of the Mediator complex [3–7]. This early modification releases Pol II from the promoter-bound preinitiation complex [6]. Afterwards, the CDK9 kinase of the positive transcription elongation factor b (P-TEFb) complex phosphorylates the Ser2 residues of the CTD, facilitating transcription elongation [5,8]. In budding yeast, the role of Ser2 phosphorylation is played by Ctk1 and Bur1, two CDK9 homologues, Ctk1 being the primary Ser2 kinase. The loss of Ctk1 nearly abolishes Ser2 phosphorylation marks [9]. However, an *in vitro* study with human P-TEFb has revealed that it preferentially phosphorylates Ser5 over other residues [10]. This discrepancy was addressed in a later study showing that Tyr1 phosphorylation directs the kinase activity of P-TEFb and alters its specificity from Ser5 to Ser2 [11]. This finding highlights that the substrate specificity of P-TEFb strongly depends on the local CTD modification context. Furthermore, P-TEFb was found to be incapable of simultaneously phosphorylating Ser2 and Ser5 of the same heptarepeat [10], further emphasizing that the local phosphorylation state influences substrate recognition.

Beyond which residues are phosphorylated, the spatial pattern and order of CTD phosphorylation events may critically influence transcriptional regulation. Previous studies suggest that P-TEFb phosphorylates the CTD distributively rather than processively, based on time-resolved mass spectrometry distributions from hyperphosphorylation assays with human [10] and *Drosophila melanogaster* [12] P-TEFb. Yet, this conclusion was based on visual inspection and was never quantitatively confirmed, e.g., via mathematical modelling. Furthermore, it remains unexplored whether already-phosphorylated sites affect the phosphorylation rate of nearby sites, either enhancing or inhibiting further modifications through altered local substrate accessibility [10], or whether P-TEFb exhibits a directional preference along the CTD. Interestingly, kinetic measurements with partially phosphorylated CTD substrates have suggested that P-TEFb may preferentially phosphorylate toward the N-terminus [10], hinting that existing modifications or structural features near the C-terminus could influence the direction of phosphorylation progression.

To address these questions, we formulate and compare four alternative mechanistic models: (i) fully processive phosphorylation, (ii) uniform distributive phosphorylation, (iii) distributive phosphorylation with local cooperativity (neighbouring-site enhancement), and (iv) directionally biased phosphorylation. For each model, we fit the parameters with mass spectrometry time-course data [10] and evaluate them using model selection criteria. We also assess the uncertainty and identifiability of the models’ parameters and simulations. To our knowledge, this represents the first quantitative, model-based comparison of phosphorylation mechanisms for P-TEFb on the CTD. This quantitative framework provides insight into whether P-TEFb acts processively, distributively without context dependence, or in a context-dependent manner shaped by local phosphorylation state or directional bias. Our results provide mechanistic constraints on CTD phosphorylation dynamics that inform how spatial mark patterns may regulate Pol II activity.

## Results

### Model structure and observables

To analyse CTD phosphorylation, we develop mathematical models that represent competing hypotheses about the underlying phosphorylation mechanisms. These models describe the dynamics of the abundance of CTDs with distinct phosphorylation patterns as well as the concurrent consumption of ATP.

We assess the plausibility of the models using published mass spectrometry data by Czudnochowski et al. [10], which quantify CTD phosphorylation configurations in an *in vitro* assay. The dataset contains 36 measurements across four time points (*t*_0_ = 0, *t*_1_ = 60, *t*_2_ = 120, and *t*_3_ = 960 minutes), reporting the distribution of CTD molecules with 0–8 phosphorylated heptarepeats. Each CTD consists of eight heptarepeats, and mass spectrometry resolves the number of phosphorylated repeats, but not their exact positions. Experimental evidence suggests that, under the conditions used, phosphorylation occurs selectively at either Ser2 or Ser5, but not both simultaneously within a single repeat [10]. Based on this, we model each heptarepeat as being either phosphorylated (1) or unphosphorylated (0), without distinguishing between the two sites.

**Fig 2:**
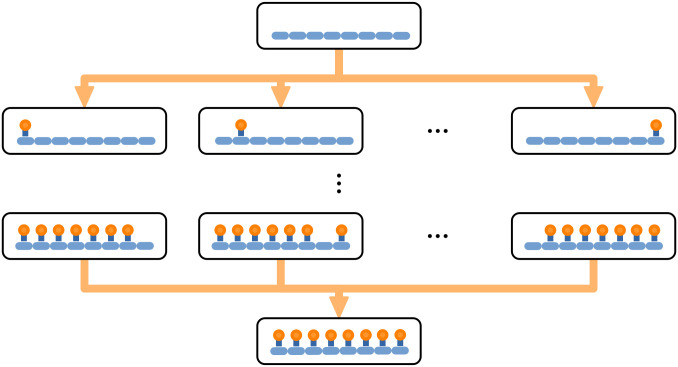
Schematic of the full model state space. Any of the eight heptarepeats (blue dashes) of the CTD molecule can be phosphorylated (orange marks) or unphosphorylated. Orange arrows indicate phosphorylation reactions.

This binary representation leads to 2^8^ = 256 possible phosphorylation configurations for heptarepeats (Fig 2). We encode the phosphorylation configuration of a heptarepeat as a vector $i\in {\left\{0,1\right\}}^{8}$, with the *r*-th vector element describing the state of the *r*-th heptarepeat (with 1 indicating phosphorylation). For instance, the configuration *i* = (0,0,1,0,1,1,0,0) corresponds to phosphorylation at repeats 3, 5, and 6. Given this encoding, we formulate a dynamical system for the time-dependent concentration of the different CTD forms, $[CTD](t)=([{CTD}_{i}](t){)}_{i\in {\left\{0,1\right\}}^{8}}$, and the concentration of ATP, [ATP](t),


$$\frac{d}{dt}\left(\begin{array}{c} \left[\mathrm{CTD}](t\right)\\ \left[\mathrm{ATP}](t\right) \end{array}\right)=\left(\begin{array}{c} f([\mathrm{CTD}](t),[\mathrm{ATP}](t),\theta )\\ g([\mathrm{CTD}](t),[\mathrm{ATP}](t),\theta ) \end{array}\right),\;\left(\begin{array}{c} \left[\mathrm{CTD}](t_{0}\right)\\ \left[\mathrm{ATP}](t_{0}\right) \end{array}\right)=\left(\begin{array}{c} {\left[\mathrm{CTD}\right]}_{t_{0}}\\ {\left[\mathrm{ATP}\right]}_{t_{0}} \end{array}\right).$$

Given the experimental substrate and ATP concentrations (100 μM and 3 mM, respectively), the system is well within the large-particle-number regime where stochastic fluctuations are negligible and a deterministic ODE description based on mass-action kinetics is appropriate.

To formalise the phosphorylation dynamics, we introduce the relation i≺j if $i_{r}\leq j_{r}$ for all r=1,…,8, meaning that configuration *i* can be phosphorylated into configuration *j*. If additionally $\|j-i{\|}_{1}=1$, then *j* differs from *i* by exactly one additional phosphorylation. Each phosphorylation step is modelled as a reaction that converts a CTD molecule from configuration *i* to configuration *j* through the addition of one phosphate group, consuming one ATP molecule in the process. For example, the reaction


$$\begin{array}{c} \left(0,1,0,0,1,0,1,0)⟶(0,1,0,1,1,0,1,0\right) \end{array}$$

describes the phosphorylation of the fourth repeat. More generally, for all pairs $i,j\in {\left\{0,1\right\}}^{8}$ with i≺j and $\|j-i{\|}_{1}=1$, we include the reaction


$$\begin{array}{c} {CTD}_{i}+ATP+\text{P-TEFb}\overset{v_{i\to j}\;}{\to }{CTD}_{j}+\text{P-TEFb}, \end{array}$$

where $v_{i\to j}$ is the rate of phosphorylation. The kinase P-TEFb is assumed to act catalytically and is not consumed during the reaction, so its concentration [P-TEFb] remains constant over time. This formulation treats each phosphorylation event as a single reaction step consuming one ATP molecule. The effect of ATP depletion on the model fit and an extension to explicit ATP and ADP binding kinetics are assessed in S1 Text, Sec. 1 and Sec. 2, respectively. Furthermore, the vector field *f* follows standard mass action kinetics for all models. Specifically, the vector field for an index $i\in {\left\{0,1\right\}}^{8}$ is given by:


$$\begin{array}{cc} f_{i}([CTD](t),[ATP](t),\theta )= & \sum_{\begin{array}{c} j\in {\left\{0,1\right\}}^{8}\\ j≺i\\ \|i-j{\|}_{1}=1 \end{array}}v_{j\to i}\cdot [{CTD}_{j}](t)\cdot [ATP](t)\cdot [\text{P-TEFb}]\\ - & \sum_{\begin{array}{c} j\in {\left\{0,1\right\}}^{8}\\ i≺j\\ \|j-i{\|}_{1}=1 \end{array}}v_{i\to j}\cdot [{CTD}_{i}](t)\cdot [ATP](t)\cdot [\text{P-TEFb}], \end{array}$$

where the first sum captures the inflow from less phosphorylated configurations, while the second sum represents the outflow to more phosphorylated configurations. Additionally, the function *g* for the time evolution of ATP is given by:


$$\begin{array}{c} g([CTD](t),[ATP](t),\theta )=-\sum_{\begin{array}{c} i,j\in {\left\{0,1\right\}}^{8}\\ i≺j\\ \|j-i{\|}_{1}=1 \end{array}}v_{i\to j}\cdot [{CTD}_{i}](t)\cdot [ATP](t)\cdot [\text{P-TEFb}]. \end{array}$$

The four model hypotheses differ in the way phosphorylation rates $v_{j\to i}$ are defined. For example, in the uniform model, all rates are equal to the base phosphorylation rate $v_{j\to i}(\theta )=k_{p}$.

In addition to the base phosphorylation rate, the model includes several observational parameters required to link the model to the experimental data. Because mass spectrometry reports relative signal intensities rather than absolute molecule concentrations, we incorporate scaling parameters into the model to ensure comparability between predictions and measurements. The full parameter vector is given by:


$$\begin{array}{c} \theta =(k_{p},s_{t_{0}},\xi_{t_{1}},\xi_{t_{2}},\xi_{t_{16}},\sigma ), \end{array}$$

which captures both dynamical and observational aspects of the system. As already mentioned, the parameter $k_{p}$ represents the base phosphorylation rate of P-TEFb under a context-independent uniform assumption. The parameter $s_{t_{0}}$ acts as a reference scaling factor, mapping the state variables of the model to the observed intensities at the initial measurement (*t*_0_). Then, since the total substrate concentration is conserved but the raw signal intensities do not sum to the same value across time points, we introduce time-point-specific scaling factors $\xi_{t_{k}}$ to account for this systematic variation, defining the effective scaling at each time point as $s_{t_{k}}=s_{t_{0}}\cdot \xi_{t_{k}}$ for k∈{1,2,3}. The standard deviation at each time point is also scaled using these factors, resulting in time-point-specific standard deviations $\sigma_{t_{k}}=\sigma \cdot \xi_{t_{k}}$, reflecting the assumption that measurement noise scales proportionally with signal intensity. In more complex hypotheses, we extend this parameter vector by including local-context sensitivity factors, such as neighbour or directional enhancement terms.

The observable mapping that relates predicted concentrations of CTDs with exactly ℓ (ℓ=0,…,8) phosphorylated repeats to the mass spectrometry measurements for a time point with index k∈{0,1,2,3} is given by:


$$\begin{array}{c} y_{\ell }(t_{k},\theta ,s_{k})=h_{\ell }([CTD](t_{k}),\theta ,s_{k})=s_{t_{k}}\cdot \sum_{\begin{array}{c} i\in {\left\{0,1\right\}}^{8}\\ \|i{\|}_{1}=\ell \end{array}}[{CTD}_{i}](t_{k}),\;\ell =0,\ldots ,8. \end{array}$$ (1)

The sum is taken over all binary configurations *i* with exactly ℓ entries equal to one.

### Model selection points to enhanced local phosphorylation

The mechanism of CTD phosphorylation has generally been considered distributive rather than processive. To evaluate this quantitatively and to test whether local inter-repeat context further modulates phosphorylation, we fit and compare three models that encode (i) a *Fully processive* mechanism, (ii) a *Uniform distributive* mechanism, and (iii) a *Neighbouring-effect* mechanism. All of these models share the same observational model described above.

**Fig 3:**
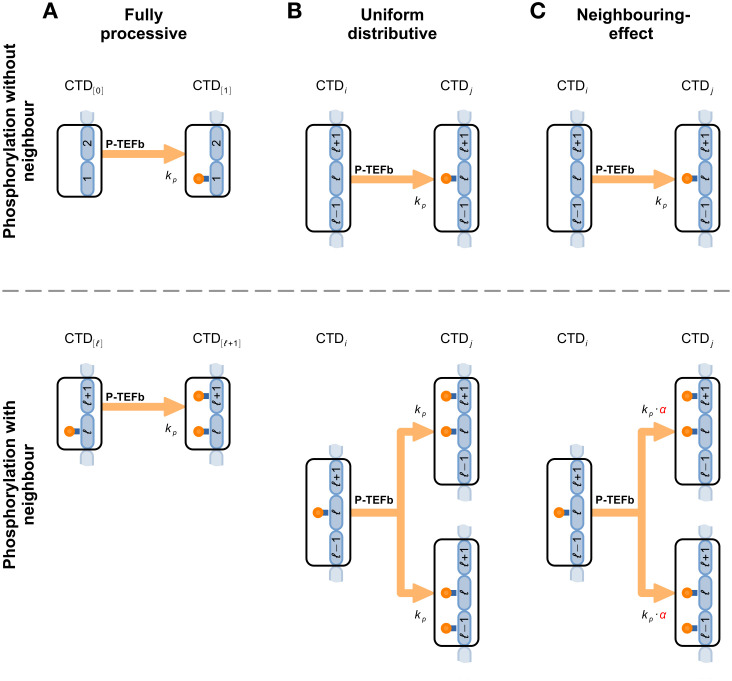
Assumptions of three model alternatives. Each schematic shows a two- (ℓ, ℓ+1) or three-repeat window (ℓ−1, ℓ, ℓ+1) within the full CTD chain (indicated by partial repeats at the top and bottom of each box). The top row depicts phosphorylation events and their rates at site ℓ when no neighbouring repeat is phosphorylated, and the bottom row when a neighbouring repeat is already phosphorylated. **(A)** Assumptions of the *Fully processive model*: P-TEFb phosphorylates heptarepeats processively, starting from one end and continuing until the other. **(B)** Assumptions of the *Uniform distributive model*: P-TEFb can phosphorylate any unphosphorylated heptarepeat with identical phosphorylation rate. **(C)** Assumptions of the *Neighbouring-effect model*: P-TEFb exhibits different phosphorylation rates for heptarepeats neighbouring already-phosphorylated heptarepeats, encoded by the multiplicative rate factor α.

In the ***Fully processive model***, phosphorylation is initiated at one end of the CTD chain and then sequentially propagates towards the other end with a phosphorylation rate $k_{p}$ (Fig 3A). Processive phosphorylation could initiate at either end of the CTD chain. This would result in a reduced state space of 16 possible phosphorylation configurations. However, since the phosphorylation rate is assumed to be constant and equal for all reactions, phosphorylation started at either end would be completely symmetric. Therefore, we consider only one direction to reduce the state space even further to 9 possible phosphorylation configurations $\overset{8}{\underset{l=0}{\left\{{CTD}_{\left[\ell \right]}\right\}}}$, where $[\ell ]\in {\left\{0,1\right\}}^{8}$ denotes the multi-index for which only the first ℓ entries are set to 1. This reduced system results in equivalent phosphorylation dynamics to the system with 16 phosphorylation configurations. For this system with 9 possible configurations, the only non-zero kinetic rates of the model are


$$\begin{array}{c} v_{\left[\ell -1]\to [\ell \right]}=k_{p}\;\text{ for }\;\ell =1,\ldots ,8. \end{array}$$

Furthermore, in a simpler case where ATP concentrations can be assumed to be approximately constant, the already-reduced system of 9 ODEs can be solved analytically (see S1 Text, Sec. 3). However, since assuming constant ATP worsens the model fit (see S1 Text, Sec. 1), we do not use this simplification for model selection.

In the ***Uniform distributive model***, any unphosphorylated repeat can be modified independently at the same rate (Fig 3B). The reaction rate between two phosphorylation configurations *i* and *j* is equal to $k_{p}$ if they differ in the phosphorylation status of a single heptarepeat, i.e.,


$$\begin{array}{c} v_{i\to j}=k_{p}\;\text{for}\;\|j-i{\|}_{1}=1. \end{array}$$

In this model, all 2^8^ = 256 phosphorylation configurations are possible. However, as all heptarepeats are phosphorylated at the same rate $k_{p}$, any phosphorylation configuration $i\in {\left\{0,1\right\}}^{8}$ with ℓ phosphorylations can be shown to have the same probability of occurrence. Namely, for any phosphorylation configuration $i\in {\left\{0,1\right\}}^{8}$ the probability of randomly choosing a CTD with phosphorylation configuration *i* from the pool of all CTDs at a given time is given by:


$$\begin{array}{c} p\left({CTD}_{i}|\tilde{P}\right)={\tilde{P}}^{\|i{\|}_{1}}(1-\tilde{P}{)}^{8-\|i{\|}_{1}} \end{array}$$

in which $\tilde{P}$ denotes the overall probability that any randomly chosen heptarepeat is phosphorylated. This probability $\tilde{P}$ can be computed by solving an ODE system with only two state variables. Furthermore, this ODE system can be solved analytically (see Materials and Methods).

In the ***Neighbouring-effect model***, the uniform rates are scaled when the newly modified site is adjacent to an already-phosphorylated repeat (Fig 3C): if ℓ denotes the phosphorylated position, then


$$\begin{array}{c} v_{i\to j}=\left\{ \begin{array}{cc} k_{p}\cdot \alpha , & \text{if }i_{\ell -1}=1\text{ or }i_{\ell +1}=1\\ k_{p}, & \text{otherwise,} \end{array}\right. \end{array}$$

with boundary sites treated as unoccupied ($i_{0}=i_{9}=0$). This breaks phosphorylation independence between sites and requires numerical integration of the full ODE systems with 2^8^ state variables.

**Fig 4:**
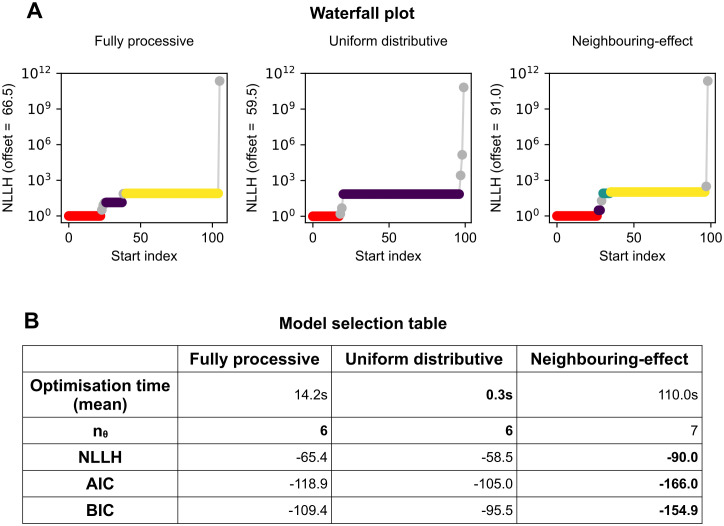
Overview of the performance of three model variants. The comparison includes the *Fully processive model*, the *Uniform distributive model*, and the *Neighbouring-effect model*. **(A)** The waterfall plots indicate the sorted values of the final NLLH values of all optimisation starts (i.e., local optimisations). **(B)** Model selection table comparing optimisation times, number of parameters ($n_{\theta }$), NLLH, AIC and BIC. The optimisation time shows the average time to execute one local optimisation of the model. Lower values of the NLLH, AIC and BIC indicate better model performance. Model selection criteria (AIC and BIC) penalise increased model complexity, i.e., larger number of parameters $n_{\theta }$.

All three models are fit to the same mass spectrometry time-course data (Fig 1B) using identical parameter bounds (see S1 Table) and multi-start optimisation. Model performance is evaluated using the Negative Log-likelihood (NLLH), Akaike Information Criterion (AIC) and Bayesian Information Criterion (BIC). Convergence and runtime are assessed using waterfall plots and per-start computation times (Fig 4).

The waterfall plots indicate good optimisation convergence for all models, with more than 10% of optimisation starts reaching the optimal value of the objective function. To further confirm that these optima are global, we repeated the optimisation using the gradient-free SaCeSS optimiser [13], which converged to the same NLLH values for all three models (see S1 Text, Sec. 4). Furthermore, as expected from the reduced state space of the *Fully processive model* and the analytical solution of the *Uniform distributive model*, they are computationally ≈8−370 times faster than the *Neighbouring-effect model*. The *Neighbouring-effect model* remains computationally feasible for the current model with 8 phosphorylation sites.

Surprisingly, the *Fully processive model* aligns better with the *in vitro* data than the *Uniform distributive model*, as reflected by lower NLLH and improved AIC/BIC scores. The *Neighbouring-effect model* further improves the fit, with a substantial reduction in both the NLLH and the model selection criteria (ΔAIC,ΔBIC>10). We also considered a *Global-effect model*, in which any existing phosphorylation globally enhances the rate of all subsequent modifications (see S1 Text, Sec. 5). Despite having the same number of parameters as the *Neighbouring-effect model*, it performs substantially worse, further supporting a local rather than global cooperativity mechanism. A comparison of the best-fit simulations for all model variants is provided in S1 Text, Sec. 6. Taken together, the results suggest that neither a purely processive nor a purely distributive mechanism can fully explain the observed *in vitro* dynamics. Hence, the *in vitro* data by Czudnochowski et al. [10] indicate the presence of a local phosphorylation enhancement effect.

These results motivate a closer examination of the *Neighbouring-effect model*, including parameter and state variable uncertainties. Subsequently, we explore whether the inclusion of directional preference of phosphorylation further improves model performance.

### *Neighbouring-effect model* provides a good data description with low uncertainty

Compared to simpler processive and distributive models, the *Neighbouring-effect model* is preferred by all model selection criteria. However, to judge whether the model is an adequate mechanistic description of the underlying phosphorylation mechanism, it is important to examine the adequacy of the fit (including residual diagnostics), test whether a more detailed enzymatic description improves the fit, and quantify the uncertainty in parameter estimates and phosphorylation dynamics.

**Fig 5:**
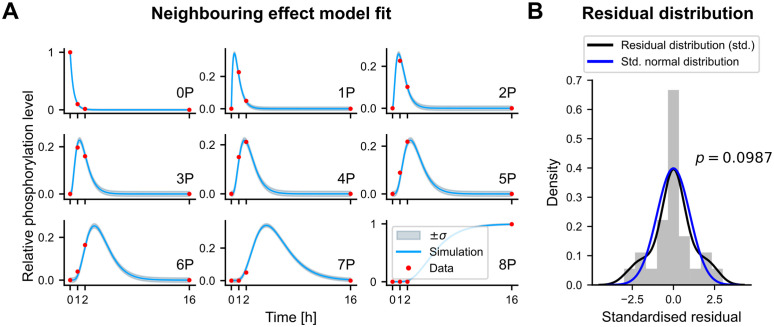
Fit of the *Neighbouring-effect model* and residual diagnostics. **(A)** Best-fit simulations (solid blue) with estimated ±σ bands (gray) versus measurements (red points). The phosphorylation count is indicated with 0P-8P. **(B)** The histogram of the standardised residuals (gray bars) and its empirical KDE distribution (black line) is compared to the standard normal distribution (blue line).

The *Neighbouring-effect model* reproduces the time-course data across phosphorylation counts (0P–8P) with close agreement between simulations and observations (Fig 5A). The model captures the early depletion of 0P and the transient accumulation of CTD concentrations of intermediate phosphorylation counts before complete phosphorylation of all CTDs. Consistent with this close agreement, the inferred measurement noise σ of the model is small.

To assess the assumption of an additive normal noise model, we compare the standardised residuals $(y_{obs}-y_{sim})/\sigma$ and their empirical KDE distribution to the standard normal distribution N(0,1) (Fig 5B). The residuals are approximately symmetric and centred at zero. Furthermore, a Shapiro-Wilk test returns *p* = 0.0987. Hence, the residuals appear to be approximately normally distributed.

**Fig 6:**
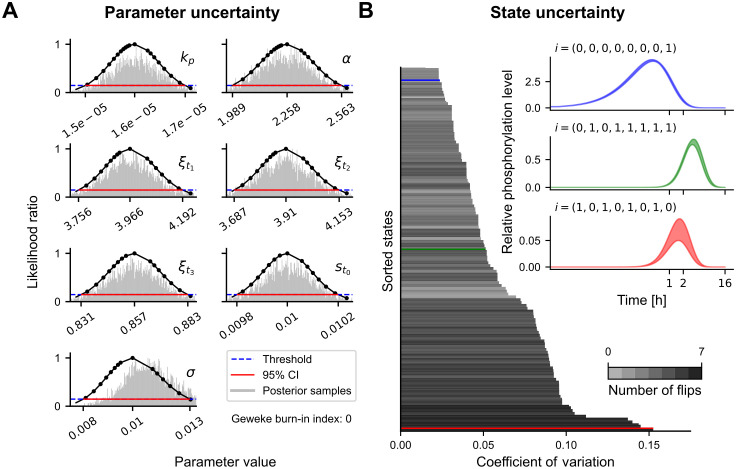
Parameter profiles and state variable uncertainty of the *Neighbouring-effect model.* **(A)** Likelihood-ratio profiles (black curves) for kinetic and observation parameters ($k_{p}$, α, $\xi_{t1}$, $\xi_{t2}$, $\xi_{t16}$, $s_{t0}$, σ) with the likelihood-ratio threshold (blue dashed line) and 95% confidence intervals (red lines). Gray histograms show the marginal posterior distributions obtained via MCMC sampling. **(B)** Uncertainty of the 256 phosphorylation configurations summarised by the coefficient of variation. The variability of all state variables is sorted and shown in gray bars. Phosphorylation configurations with a more alternating pattern (more 0-1 and 1-0 flips) are shown in darker gray, and simpler configurations with less alternation are shown in lighter gray. To connect the coefficient of variation to uncertainty of the state trajectory, we present three examples with lower (blue), medium (green), and larger (red) coefficient of variation.

To explore how well the data identifies all kinetic and observational model parameters, we compute profile likelihoods of all parameters (Fig 6A). Each parameter profile is uni-modal and crosses the likelihood‑ratio threshold on both sides of the optimum, yielding narrow confidence intervals. This is evidence of practical identifiability and low parameter uncertainty. Notably, the neighbouring‑effect parameter α is tightly bounded and its confidence interval remains above 1, indicating a confident local enhancement, rather than a reduction, of phosphorylation adjacent to already-phosphorylated sites. The *Fully processive model* and the *Uniform distributive model* give rise to equally narrow and uni-modal parameter profiles (see S1 and S2 Figs). As a complementary assessment, we performed MCMC sampling (see Materials and Methods) and overlaid the marginal posterior distributions on the profile likelihoods (gray histograms in Figs 6A, S1, and S2). The two approaches show close agreement for all parameters, confirming the robustness of the uncertainty estimates. Minor differences arise for parameters with correlations (e.g., σ and $\xi_{t_{k}}$).

Having established that all parameters are identifiable, we next tested whether the single-step phosphorylation formulation is an adequate level of mechanistic detail. Extending the *Neighbouring-effect model* to include explicit ATP binding, phosphotransfer, and ADP release (see S1 Text, Sec. 2) does not improve the fit according to AIC and BIC, while the additional kinetic parameters are largely non-identifiable. The local enhancement factor α remains consistent across all variants (α≈2.1−2.4), confirming that the cooperativity finding is robust to the level of enzymatic detail.

Lastly, we inspect how parameter uncertainty propagates through model simulation and how it affects the uncertainty in unobserved model state variables. To this end, we collect an ensemble of parameter vectors obtained by uniformly sampling parameter values within the profile‑derived confidence intervals and retaining only parameter vectors that fall inside the joint confidence region. For each parameter vector in this ensemble, we simulate the model, yielding information about the uncertainty of the state trajectories. The computation of the time-aggregated coefficient of variation for each of the 256 phosphorylation configurations,


$$\begin{array}{c} \frac{\sum_{t}\mathrm{sd}[x_{s}(t)]}{\sum_{t}𝔼[x_{s}(t)]}, \end{array}$$

indicates an overall low uncertainty of the model state variables (Fig 6B). As expected, states with simple phosphorylation configurations, i.e., very low phosphorylation or near‑complete phosphorylation, show the least uncertainty. In contrast, states with alternating phosphorylation patterns with many 0–1 and 1–0 flips, e.g., (0,1,0,1,0,1,0,1), show greater uncertainty. This is due to a stronger effect of the local enhancement factor α on the dynamics of these state variables. Thus, the uncertainty in the factor α is propagated to the uncertainty in these states. However, even in these cases, the uncertainty remains moderate as can be seen in the state trajectories: the uncertainty bands remain narrow relative to the trajectory peaks (Fig 6B).

In conclusion, the *Neighbouring‑effect model* provides a good quantitative description of the data with low estimated measurement noise, identifiable parameters, and low uncertainty of unobserved model state variables.

### Data do not provide evidence of a directional phosphorylation bias

**Fig 7:**
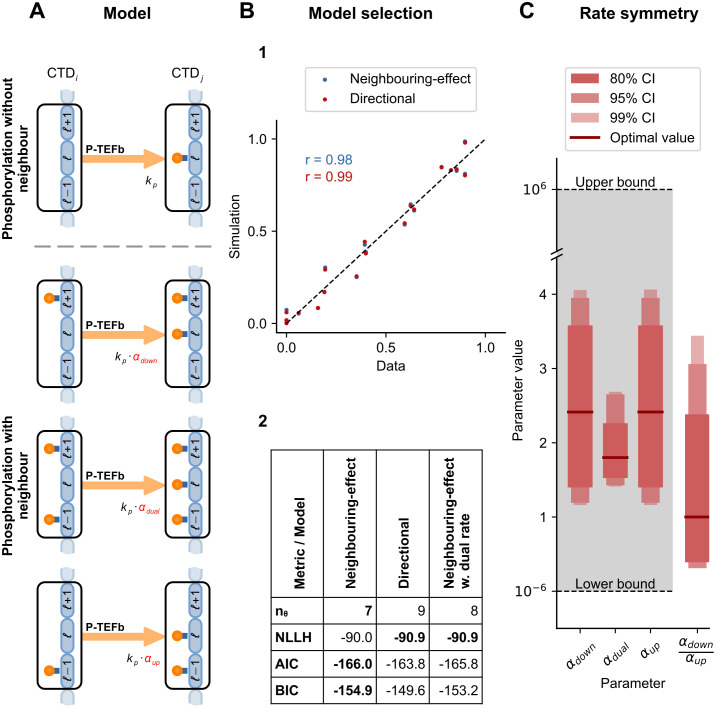
The *Directional model* and comparison with the *Neighbouring-effect model.* **(A)** Assumptions of the *Directional model*: schematic of base ($k_{p}$), upward ($k_{p}\alpha_{\text{up}}$), downward ($k_{p}\alpha_{\text{down}}$), and dual ($k_{p}\alpha_{\text{dual}}$) phosphorylation events. The top part depicts phosphorylation events and their rates at site ℓ when no neighbouring repeat is phosphorylated, and the bottom part when a neighbouring repeat is already phosphorylated. (B1) Data–simulation scatter plot comparing the fit of the *Neighbouring-effect model* and the *Directional model*. (B2) Values of the NLLH, AIC, BIC, and parameter counts for the *Neighbouring-effect model*, the *Directional model*, and the *Neighbouring-effect model* with a dual rate. **(C)** Profile-derived confidence intervals and optima of the *Directional model* for $\alpha_{\text{up}}$, $\alpha_{\text{down}}$, $\alpha_{\text{dual}}$, and the ratio $\alpha_{\text{down}}/\alpha_{\text{up}}$.

In the previous section, we established that the *Neighbouring-effect model* provides a good description of the experimental data. However, it is not clear whether the data also support a directional preference for phosphorylation along the CTD. To test this hypothesis, we extend the *Neighbouring‑effect model* to a *Directional model* by allowing for asymmetric local enhancement of phosphorylation (Fig 7A): an upward factor $\alpha_{\text{up}}$ and a downward factor $\alpha_{\text{down}}$. This model also includes a dual factor $\alpha_{\text{dual}}$ when the target phosphorylation site is flanked by phosphorylated neighbours on both sides. Formally, for a transition i→j that phosphorylates site ℓ,


$$\begin{array}{c} v_{i\to j}=\left\{ \begin{array}{cc} k_{p}\;\alpha_{\text{dual}}, & i_{\ell -1}=1\wedge i_{\ell +1}=1\\ {}[2pt]k_{p}\;\alpha_{\text{up}}, & i_{\ell -1}=1\wedge i_{\ell +1}=0\\ {}[2pt]k_{p}\;\alpha_{\text{down}}, & i_{\ell +1}=1\wedge i_{\ell -1}=0\\ {}[2pt]k_{p}, & \text{otherwise}, \end{array}\right. \end{array}$$

with boundary sites treated as unoccupied ($i_{0}=i_{9}=0$). Furthermore, for completeness, we compare this model to a *Neighbouring-effect model with dual rate* which extends the *Neighbouring-effect model* with the dual rate. The observational model and estimation procedure are identical to the previous analysis.

The predicted versus observed values overlap almost perfectly for the *Neighbouring‑effect model* and the *Directional model*, with data–simulation correlations *r* = 0.98 and *r* = 0.99, respectively (Fig 7B1). This indicates that introducing separate upward/downward rates does not change the quality of the fit to the experimental data. The *Directional model* achieves a slightly lower NLLH than the *Neighbouring‑effect model*, but the improvement is insufficient to compensate for the additional parameters; the AIC and BIC are higher for the *Directional model* than for the *Neighbouring‑effect model* (Fig 7B2). Adding only the dual rate to the *Neighbouring-effect model* in the *Neighbouring-effect model with dual rate* (i.e., without up/down asymmetry) achieves the same NLLH as the full *Directional model* but still worsens the AIC/BIC because of the extra parameter. This equality is expected: the fitting of the *Directional model* yields $\alpha_{\text{up}}^{\text{opt}}\approx \alpha_{\text{down}}^{\text{opt}}$ (Fig 7C), so the asymmetry is unused and the likelihood gain stems from the dual context alone.

As for other model parameters, the profile‑derived confidence intervals for $\alpha_{\text{up}}$, $\alpha_{\text{down}}$, and $\alpha_{\text{dual}}$ are narrow, indicating good practical identifiability (see S3 Fig for full parameter profiles). Since the optimal values for $\alpha_{\text{up}}$ and $\alpha_{\text{down}}$ are essentially equal, their ratio $\alpha_{\text{down}}/\alpha_{\text{up}}$ is tightly concentrated around the optimal value of ≈1. Notably, $\alpha_{\text{dual}}$ is estimated below both single‑neighbour factors, suggesting that a site flanked by two phosphorylated neighbours is not modified faster than one with a single neighbour under these conditions (Fig 7C).

In conclusion, within the resolution of the current *in vitro* data, there is no compelling evidence for a directional bias in CTD phosphorylation. The *Neighbouring‑effect model* remains optimal according to model selection criteria.

## Discussion

CTD phosphorylation patterns regulate the timing and coordination of transcriptional processes, which is essential for proper gene expression and cellular function. However, the mechanistic principles that govern the kinase-mediated phosphorylation of CTD sites and establish these patterns are not fully understood. Although previous studies conclude that P-TEFb acts distributively based on visual inspection of mass spectrometry data [10,12], it remains unclear whether the local phosphorylation context further shapes the phosphorylation dynamics. Here we find, through a quantitative model comparison, a strong indication that P-TEFb phosphorylates the CTD distributively with local cooperativity. Repeats adjacent to already-phosphorylated sites are modified at substantially higher rates. In contrast, we find no evidence for a directional preference of P-TEFb phosphorylation. However, this may be due to the resolution of the *in vitro* data [10] that we use for model estimation. Subsequent analysis of time-series data with individual site resolution or using pre-phosphorylated CTDs would be necessary to conclusively rule out directional bias in P-TEFb phosphorylation.

Context-dependent modulation of CTD phosphorylation has been observed in several forms, but direct evidence for inter-repeat cooperativity has been lacking. Our analysis identifies such inter-repeat effects, where phosphorylation of one heptarepeat accelerates modification of neighbouring heptarepeats. Previous studies have mainly described intra-heptad dependencies. For example, pre-phosphorylation of Ser7 increases the activity of P-TEFb towards Ser2 within the same repeat [10]. In *Saccharomyces cerevisiae*, phosphorylation of Ser5 by Kin28 promotes the recruitment and Ser2 kinase activity of the Bur1/Bur2 complex early in transcription elongation [14]. Beyond such intra-heptad dependencies, mass spectrometric mapping of CTD phosphorylation in human cells has shown that adjacent heptarepeats are preferentially co-phosphorylated at the same residue, with both Ser2 and Ser5 marks most often accompanied by the same mark in the adjacent N- or C-terminal repeat [15]. As human P-TEFb (CDK9/Cyclin T) is the kinase used in the *in vitro* assay analysed here, this in vivo neighbour-correlation pattern is consistent with the local cooperativity inferred by our model. However, not all CTD-modifying enzymes exhibit this dependence on the local context. For instance, O-GlcNAc transferase (OGT) catalyses the attachment of O-linked N-acetylglucosamine residues to the Ser and Thr sites of the CTD through a purely distributive mechanism [16]. Each modification event occurs independently of neighbouring sites, producing a heterogeneous mixture of glycoforms. This contrasts with the locally cooperative phosphorylation behaviour we observe for P-TEFb, highlighting that distributive modification of the CTD can arise with or without spatial interdependence between sites.

Our inference of local cooperativity implies that, even under a distributive mechanism, phosphorylation would tend to accumulate in short stretches of neighbouring repeats, forming clusters of phosphorylated sites. However, this conclusion is based on *in vitro* time-course data with resolution limited to the total number of phosphorylated repeats rather than their specific positions. Consequently, the presence and extent of such clustering *in vivo* remain open questions and should be further explored using approaches capable of resolving CTD modification patterns at or near the level of individual repeats over time. Clear observation of transient, neighbour-to-neighbour enrichment would provide stronger evidence for inter-heptad cooperativity. Similarly, the molecular mechanism underlying the observed cooperativity remains an open question. The enhancement factor α may reflect conformational changes in the CTD upon phosphorylation, altered accessibility of neighbouring repeats, or increased local kinase concentration through rebinding. The local enhancement captured by α is also consistent with a short-range processive mechanism, in which the kinase modifies a few adjacent sites before detaching. A strictly directional variant of such a mechanism is not supported by the data, as the *Directional model* (Fig 7) finds no evidence for asymmetric enhancement. Distinguishing between these possibilities would require targeted experimental approaches beyond the scope of the current *in vitro* assay.

The level of mechanistic detail in the models was chosen to match the resolution of the available data. Each phosphorylation event is treated as a single reaction step, and as shown in the Results, extending this to explicit ATP and ADP binding kinetics does not improve the description. More broadly, the current model represents a deliberately simplified *in vitro* setting, designed to isolate the kinetic mechanism of P-TEFb phosphorylation on a short synthetic CTD substrate. *In vivo*, CTD phosphorylation involves multiple kinases and phosphatases acting in a temporally coordinated manner throughout the transcription cycle, and such interactions are not captured here. Extending this modelling framework to include additional kinases would represent a natural next step.

However, even within the *in vitro* context, expanding the model to longer CTDs introduces a severe computational challenge: the number of possible phosphorylation configurations increases exponentially with the number of repeats ($2^{n}$). Such combinatorial explosion of molecular states is a general challenge in biochemical modelling. Reduction strategies have been proposed to address this, including replacing micro-states by macro-descriptions of independent binding domains [17], automatic identification of independent molecular fragments for coarse-graining within rule-based modelling frameworks [18,19], and more general approaches based on time-scale separation [20]. The reductions we derive for the processive and distributive models similarly exploit the independence of phosphorylation sites to reduce the state space from $2^{n}$ to *n* + 1 or fewer variables. By contrast, the *Neighbouring-effect model* introduces local dependencies between sites, which breaks the independence required for such reductions. Developing reduced or analytical formulations for models with local interactions would be key to scaling the approach to realistic CTD lengths. More broadly, this modelling framework could be adapted to study other multisite modification systems, such as histone or RNA tail phosphorylation, where local context may similarly shape phosphorylation dynamics. Furthermore, incorporating opposing phosphatases would enable analysis of steady-state phosphorylation distributions, while connecting the predicted phosphorylation patterns to functional transcriptional readouts [21] would strengthen the biological interpretation of the inferred cooperativity.

In summary, our analysis reveals that P-TEFb phosphorylates the CTD distributively but with strong local cooperativity, such that phosphorylation of one repeat enhances modification of its neighbours. This finding highlights that local context can significantly influence the dynamics of CTD phosphorylation. The model developed here offers a minimal yet extensible framework for dissecting multisite modification dynamics and can serve as a foundation for future studies incorporating additional kinases, regulatory interactions, or longer CTD constructs.

## Materials and methods

### Experimental setup and measurement

We analysed phosphorylation data from the *in vitro* experiments of Czudnochowski et al. [10], which examined how P-TEFb phosphorylates the RNAP II CTD. Mass spectrometry (ESI-MS) assays were performed to determine the distribution of phosphorylation configurations on the CTD peptides. For early time points (1 and 2 hours, in addition to the 0-hour baseline), a synthetic peptide comprising eight CTD repeats (CTD [8], without a GST tag) was used as substrate. At the final 16-hour time point, a GST-tagged eight-repeat CTD (GST–CTD [8]) was employed to improve ionization efficiency. In both cases, the CTD peptide concentration was 100 μM, incubated with 0.1 μM P-TEFb and 3 mM non-radioactive ATP in kinase buffer (50 mM HEPES pH 7.6, 34 mM KCl, 7 mM MgCl_2_, 2.5 mM DTT, 5 mM β-glycerol phosphate, 0.5 mM Na${\;}_{3}{\text{VO}}_{4}$) at $30^{\circ }$C. ESI-MS readouts provided relative abundances of CTD molecules with phosphorylation counts ranging from 0 to 8.

### Analytical solution for the *Uniform distributive model*

Since the *Uniform distributive model* assumes no cross-interactions between CTD repeats and that all repeats follow identical kinetics, the total concentration of phosphorylated repeats across all CTD chains can be represented by a single reaction:


$$\begin{array}{c} U+ATP+\text{P-TEFb}\overset{k_{p}\;}{\to }P+\text{P-TEFb}, \end{array}$$ (2)

where *U* and *P* denote the total concentrations of unphosphorylated and phosphorylated repeats, respectively. To ensure these variables represent total repeat concentrations, the initial value of *U* is set to the total substrate concentration scaled by the number of repeats per CTD chain, i.e., $U_{0}=8\cdot S$. Additionally, the total substrate concentration is conserved over time, so we can represent the concentration of the unphosphorylated repeats as $U=U_{0}-P$. Thus, reaction (2) gives rise to the following ODE system with a conserved quantity:


$$\begin{array}{cc} \frac{d[P]}{dt} & =k_{p}[U]\cdot [\text{ATP}]\cdot [\text{P-TEFb}]\\ \frac{d[\text{ATP}]}{dt} & =-k_{p}[U]\cdot [ATP]\cdot [\text{P-TEFb}]\\ \;[U] & =U_{0}-[P]. \end{array}$$

Furthermore, with $\tilde{U}=[U]/U_{0}$ we represent the fraction of unphosphorylated repeats and with $\tilde{P}=[P]/U_{0}$ the fraction of phosphorylated repeats. Consequently, these fractions satisfy $\tilde{U}=1-\tilde{P}$. To solve the system analytically, we first note that $\dot{\left[P\right]}+\dot{\left[\text{ATP}\right]}=0$, which implies that


$$[\text{ATP}](t)={\text{ATP}}_{0}-[P](t).$$

For notational simplicity, we assume that the constant factor [P-TEFb] is absorbed into $k_{p}$. Substituting the algebraic equation for [ATP] into the first ODE yields,


$$\begin{array}{c} \frac{d[P]}{dt}=k_{p}(U_{0}-[P])({\text{ATP}}_{0}-[P]). \end{array}$$ (3)

When ${\text{ATP}}_{0}=U_{0}$, equation (3) simplifies to


$$\begin{array}{c} \frac{d[P]}{dt}=k_{p}(U_{0}-[P]{)}^{2}, \end{array}$$

which can be solved by separation of variables to obtain


$$\begin{array}{c} P(t)=U_{0}-\frac{U_{0}}{1+U_{0}\cdot k_{p}\cdot t}. \end{array}$$

For ${\text{ATP}}_{0}\text{≠}U_{0}$, we rewrite equation (3) as


$$\begin{array}{c} \frac{1}{\left(U_{0}-[P])({\text{ATP}}_{0}-[P]\right)}\cdot \frac{d[P]}{dt}=k_{p}. \end{array}$$

Using partial fraction decomposition, the left term becomes


$$\begin{array}{c} \frac{1}{{\text{ATP}}_{0}-U_{0}}\left(\frac{1}{U_{0}-[P]}-\frac{1}{{\text{ATP}}_{0}-[P]}\right)\cdot \frac{d[P]}{dt}=k_{p}. \end{array}$$

Integrating both sides and applying the initial condition [*P*](0) = 0 yields


$$\begin{array}{c} \frac{1}{{\text{ATP}}_{0}-U_{0}}\ln\left(\left|\frac{{\text{ATP}}_{0}-[P]}{U_{0}-[P]}\right|\right)=k_{p}\cdot t+\frac{1}{{\text{ATP}}_{0}-U_{0}}\ln\left(\left|\frac{{\text{ATP}}_{0}}{U_{0}}\right|\right). \end{array}$$

Solving for *P* gives


$$\begin{array}{c} P(t)=\frac{{\text{ATP}}_{0}\cdot U_{0}\left(e^{{\text{ATP}}_{0}\cdot k_{p}\cdot t}-e^{U_{0}\cdot k_{p}\cdot t}\right)}{{\text{ATP}}_{0}\cdot e^{{\text{ATP}}_{0}\cdot k_{p}\cdot t}-U_{0}\cdot e^{U_{0}\cdot k_{p}\cdot t}}. \end{array}$$

Combining both cases, we obtain the following closed-form solution:


$$\begin{array}{c} P(t)=\left\{ \begin{array}{c} U_{0}-\frac{U_{0}}{1+U_{0}\cdot k_{p}\cdot t}\;\text{if }{\text{ATP}}_{0}=U_{0}\\ \frac{{\text{ATP}}_{0}\cdot U_{0}\left(e^{{\text{ATP}}_{0}\cdot k_{p}\cdot t}-e^{U_{0}\cdot k_{p}\cdot t}\right)}{{\text{ATP}}_{0}\cdot e^{{\text{ATP}}_{0}\cdot k_{p}\cdot t}-U_{0}\cdot e^{U_{0}\cdot k_{p}\cdot t}}\;\text{otherwise.} \end{array}\right. \end{array}$$ (4)

Now, to determine the temporal evolution of the distribution of CTD configurations, we first note that there are no cross-interactions between repeats and all phosphorylation events occur with identical kinetics. Therefore, the phosphorylation configurations of individual repeats are statistically independent. Consequently, the concentration of CTD chains exhibiting a specific phosphorylation pattern $i\in {\left\{0,1\right\}}^{8}$ can be expressed as the product of the fractions associated with each repeat being in its respective phosphorylation configuration, scaled by the total substrate concentration. Hence,


$$[{CTD}_{i}]=S\cdot {\tilde{P}}^{\|i{\|}_{1}}\cdot {\left(1-\tilde{P}\right)}^{8-\|i{\|}_{1}}.$$

For the parameter estimation, the concentration of specific phosphorylation patterns is not relevant but only the overall concentration of CTDs with a specific number of phosphorylations, which is given by:


$$\begin{array}{c} \begin{array}{cc} \sum_{\begin{array}{c} i\in {\left\{0,1\right\}}^{8}\\ \|i{\|}_{1}=\ell \end{array}}[{CTD}_{i}] & =\sum_{\begin{array}{c} i\in {\left\{0,1\right\}}^{8}\\ \|i{\|}_{1}=\ell \end{array}}S\cdot {\tilde{P}}^{\|i{\|}_{1}}\cdot {\left(1-\tilde{P}\right)}^{8-\|i{\|}_{1}}\\ & =S\cdot \sum_{\begin{array}{c} i\in {\left\{0,1\right\}}^{8}\\ \|i{\|}_{1}=\ell \end{array}}{\tilde{P}}^{\ell }\cdot {\left(1-\tilde{P}\right)}^{8-\ell } \end{array}. \end{array}$$

Since choosing a phosphorylation pattern with ℓ phosphorylated repeats amounts to selecting ℓ out of 8 sites, the total number of such configurations is (8ℓ). Thus, the concentration of CTD chains with exactly ℓ phosphorylated repeats follows a binomial distribution with parameters *n* = 8 and time-dependent success probability $\tilde{P}$, scaled by *S*:


∑i∈{0,1}8‖i‖1=ℓ[CTDi]=S·(8ℓ)P~ℓ·(1−P~)8−ℓ.

This shows that, instead of explicitly modelling all 2^8^ possible phosphorylation configurations, it is sufficient to track the total phosphorylation dynamics. The full distribution of CTD configurations can then be directly inferred via this binomial relationship. The model observables can then be written as


yℓ(tk,θ)=stk·S·(8ℓ)P~ℓ(tk)·(1−P~(tk))8−ℓ.

Together with the analytical solution for *P*, and thus $\tilde{P}$, the observables can be calculated entirely analytically.

### Numerical model simulation

Time-course simulations of the ODE models were performed using the CVODES solver from the SUNDIALS suite, through AMICI’s interface [22]. CVODES provides efficient and accurate integration of stiff systems using adaptive step-size and order control, ensuring numerical stability across a wide range of model dynamics. Simulations were performed with a relative tolerance of 10^-7^ and an absolute tolerance of 10^-16^.

### Model parametrisation

All models in this manuscript are parametrised using maximum likelihood estimation. We assume additive and normally distributed measurement noise. Thus, the relation of the model observables (1) with exactly ℓ (=0,…,8) phosphorylated repeats at a time point with index k∈{0,1,2,3} to the respective observed mass spectrometry data point $y_{\ell ,t_{k}}^{\text{obs}}$ is given by:


$$\begin{array}{c} y_{\ell ,t_{k}}^{\text{obs}}=y_{\ell }(t_{k},\theta )+\varepsilon =h_{\ell }([CTD](t_{k}),\theta )+\varepsilon ,\;\varepsilon \sim 𝒩(0,\sigma_{k}^{2}). \end{array}$$

The conditional probability density of observing a specific data point at a specific time point given the model observable $y_{\ell }(t_{k},\theta ,s_{k})$ and noise parameter $\sigma_{k}$ is given by:


$$\begin{array}{c} p(y_{\ell ,t_{k}}^{\text{obs}}|y_{\ell }(t_{k},\theta ,s_{k}),\sigma_{k})=\frac{1}{\sqrt{2\pi }\sigma_{k}}\exp\left[-\frac{{\left(y_{\ell ,t_{k}}^{\text{obs}}-y_{\ell }(t_{k},\theta ,s_{k})\right)}^{2}}{2\sigma_{k}^{2}}\right]. \end{array}$$

We assume all measurements are mutually independent, so the likelihood function is given as a product of conditional probabilities:


$$\begin{array}{c} ℒ(\theta ,s,\sigma )=\prod_{\ell =0}^{8}\prod_{k=0}^{3}p(y_{\ell ,t_{k}}^{\text{obs}}|y_{\ell }(t_{k},\theta ,s_{k}),\sigma_{k}). \end{array}$$

As is commonly done, instead of maximising the likelihood function, for better numerical stability, we minimise the negative log-likelihood, which is given by:


$$\begin{array}{c} J(\theta ,s,\sigma )=-\log ℒ(\theta ,s,\sigma )=\sum_{\ell =0}^{8}\sum_{k=0}^{3}\log(2\pi \sigma_{k})+\left(\frac{(y_{\ell ,t_{k}}^{\text{obs}}-y_{\ell }(t_{k},\theta ,s_{k}){)}^{2}}{2\sigma_{k}^{2}}\right). \end{array}$$

We define the maximum likelihood estimate as the minimum of this objective function


$$\begin{array}{c} \hat{\theta }={\mathrm{argmin}}_{\theta }J(\theta ). \end{array}$$

We minimise the objective function using multi-start minimisation with 128 local minimisations per model. For this, we employ gradient-based optimisation using the trust region optimiser Fides [23]. Gradients of the objective function are computed analytically for analytical models and computed via adjoint sensitivity analysis using AMICI [22] for all other models. Adjoint sensitivity analysis was performed with a relative tolerance of 10^-7^ and an absolute tolerance of 10^-16^.

### Model selection criteria

To compare the performance of different models in a principled way, we employ the **Akaike Information Criterion** (AIC) [24] and the **Bayesian Information Criterion** (BIC) [25]. Both measures aim to balance goodness-of-fit with model complexity, penalising models that use more parameters to prevent overfitting. The AIC is defined as


$$\begin{array}{c} \text{AIC}=2J(\hat{\theta })+2n_{\theta }, \end{array}$$

where $n_{\theta }$ denotes the number of parameters in the model and *J* the negative log-likelihood. Therefore, models that achieve a good fit (low NLLH) will be rewarded while excessive model complexity (high $n_{\theta }$) will be penalised. Thus, in general a lower AIC value indicates a more favourable trade-off between model fit and model complexity. The BIC extends this idea by also incorporating the number of data points *n*_data_:


$$\begin{array}{c} \text{BIC}=2J(\hat{\theta })+n_{\theta }\ln(n_{\text{data}}). \end{array}$$

### Uncertainty quantification

We express the uncertainty of parameter estimates using their respective confidence intervals. We compute these intervals using the profile likelihood approach based on the likelihood-ratio test. The likelihood-ratio test for a parameter vector θ is defined via the test statistic:


$$\begin{array}{c} \Lambda (\theta )=-2\ln\left(\frac{L(\theta )}{ℒ(\hat{\theta })}\right)=2(J(\theta )-J(\hat{\theta })), \end{array}$$

where ℒ denotes the likelihood function. In the asymptotic case of a large number of data points, the distribution of Λ(θ) can be approximated by the chi-square distribution $\chi_{n_{\theta }}^{2}$ with degrees of freedom equal to the number of parameters [26]. The confidence region of significance level α is defined as


$$\begin{array}{c} {CR}^{\alpha }=\{\theta |\Lambda (\theta )\leq \Delta_{\alpha }(n_{\theta })\}=\left\{\theta |J(\theta )\leq J(\hat{\theta })+\frac{\Delta_{\alpha }(n_{\theta })}{2}\right\}, \end{array}$$

where $\Delta_{\alpha }(n_{\theta })$ denotes the (1−α)-quantile of the $\chi_{n_{\theta }}^{2}$ distribution. For a single parameter, the profile likelihood is defined as


$$\begin{array}{c} PL(\theta_{i})=\min_{{\tilde{\theta }}_{j\neq i}}J(\tilde{\theta }). \end{array}$$

Similarly to the confidence region, the profile likelihood-based confidence interval is defined as


$$\begin{array}{c} {CI}_{\theta_{i}}=\{\theta_{i}|PL(\theta_{i})\leq J(\hat{\theta })+\Delta_{\alpha }(1)\}. \end{array}$$

The profile likelihood provides not only a numerical estimate of parameter uncertainty through the confidence interval width, but also a visual and qualitative assessment of how sensitively the likelihood responds to changes in each parameter. Furthermore, we assess practical identifiability using profile-derived confidence intervals: a combination of model and data is said to be practically identifiable if the confidence intervals of all parameters are finite.

To complement the frequentist uncertainty quantification provided by profile likelihoods, we perform Bayesian uncertainty analysis. We consider log-uniform priors between the parameter bounds and generate posterior samples using Markov chain Monte Carlo (MCMC) sampling with the adaptive Metropolis algorithm [27]. For each model, 10^5^ samples are generated, initialised at the maximum likelihood estimate. The burn-in period is determined using the Geweke diagnostic, and the remaining samples are thinned based on the estimated autocorrelation length to obtain approximately independent posterior samples.

### Implementation

The model definition and parameter estimation tasks were formulated using the PEtab (v0.7.0) format [28]. Models were created with PySB (v1.16.0) [29], a rule-based modelling framework built on BioNetGen [30], and encoded in SBML [31]. Parameter estimation and uncertainty analysis were performed using pyPESTO (v0.5.7) [32]. The model simulations used AMICI (v0.34.2) [22], and the trust region optimiser Fides (v0.8.0) [23] was used for numerical optimisation.

### Declaration of usage of AI tools in the writing process

Portions of the text were refined with the assistance of ChatGPT and Claude, which was used for language editing and improving clarity. The authors reviewed and approved all generated text and are responsible for the final content.

## Supporting information

S1 Text Supplementary analyses and model variants. Comparison of best-fit simulations across all model variants, description and results of the *Global-effect model*, comparison of constant and variable ATP assumptions, analytical solution for the *Fully processive model*, extended models with detailed ATP and ADP binding mechanics, and verification of optimisation results using the gradient-free SaCeSS optimiser.
(PDF)

S1 Fig Profile likelihoods for parameters of the *Fully processive model.* Black curves show the obtained profile likelihood (maximum likelihood obtained for the fixed parameter value) normalised by the maximum likelihood from optimisation. The red line marks the 95% confidence threshold, and the blue dashed line indicates the CI cutoff. Gray histograms show the marginal posterior distributions obtained via MCMC sampling.
(PDF)

S2 Fig Profile likelihoods for parameters of the *Uniform distributive model.* Black curves show the obtained profile likelihood (maximum likelihood obtained for the fixed parameter value) normalised by the maximum likelihood from optimisation. The red line marks the 95% confidence threshold, and the blue dashed line indicates the CI cutoff. Gray histograms show the marginal posterior distributions obtained via MCMC sampling.
(PDF)

S3 Fig Profile likelihoods for parameters of the *Directional model.* Black curves show the obtained profile likelihood (maximum likelihood obtained for the fixed parameter value) normalised by the maximum likelihood from optimisation. The red line marks the 95% confidence threshold, and the blue dashed line indicates the CI cutoff. Gray histograms show the marginal posterior distributions obtained via MCMC sampling.
(PDF)

S1 Table Parameters of all models. Contains all parameters used in all models with their parameter bounds, confidence intervals across models, and parameter units.
(XLSX)
