# Supplementary material for: Quantitative modelling of P-TEFb mediated CTD phosphorylation identifies local cooperativity
Source: PLoS Comput Biol. 2026 Jul 30;22(7):e1014531. doi: 10.1371/journal.pcbi.1014531 (PMC13423041; doi:10.1371/journal.pcbi.1014531)
Supplement: S1 Text — Comparison of best-fit simulations across all model variants, description and results of the Global-effect model, comparison of constant and variable ATP assumptions, analytical solution for the Fully processive model, extended models with detailed ATP and ADP binding mechanics, and verification of optimisation results using the gradient-free SaCeSS optimiser. (PDF) [file pcbi.1014531.s001.pdf]

Supplementary information - Quantitative modelling of P-TEFb mediated CTD phosphorylation identifies local cooperativity

Aaron Callenbach[1,2◕], Domagoj Dorešić[1,2◕], Robert Düster[3], Vanessa Nakonecnij[1], Erika Dudkin[1], Matthias Geyer[3], Jan Hasenauer[1,2*]

[1] Life and Medical Sciences (LIMES) Institute, University of Bonn, Bonn, 53115, Germany

[2] Bonn Center for Mathematical Life Sciences, University of Bonn, Bonn, 53115, Germany

[3] Institute of Structural Biology, University of Bonn, Bonn, 53127, Germany

◕ These authors contributed equally to this work.

Contents

1 Neighbouring-effect model with constant ATP **2**

2 Extended models with detailed ATP binding mechanics **2**

2.1 Reversible ATP and ADP kinetics 3

2.2 Irreversible ATP and ADP kinetics 3

2.3 Comparison 4

3 Analytical solution for the *Fully processive model* **5**

4 Global optimisation with SaCeSS **9**

5 Global-effect model **9**

6 Comparison of model fits **10**

1 Neighbouring-effect model with constant ATP

In the main text, all models include ATP as a dynamic variable that is consumed during phosphorylation. To assess whether ATP dynamics meaningfully affect the model fit, we compare the *Neighbouring-effect model* with variable ATP to a variant in which the ATP concentration is held constant throughout the simulation.

In the experimental setup of Czudnochowski et al.[2], the initial ATP concentration is 3 mM, while the total number of phosphorylatable sites is $8 \times 100\ \mu\text{M} = 0.8$ mM. If all sites are phosphorylated, ATP is reduced by approximately 27%, from 3 mM to 2.2 mM. This depletion is sufficient to affect phosphorylation rates, particularly at later time points.

The comparison shows that including ATP dynamics improves the model fit (Table 1). With the same number of parameters ($n_\theta = 7$), the variable-ATP model achieves a lower NLLH (-90.0 vs. -87.8), AIC (-166.0 vs. -161.6), and BIC (-154.9 vs. -150.5) than the constant-ATP variant. While the differences are moderate, including ATP depletion is the appropriate choice given the experimental conditions, where ATP is reduced by approximately 27% upon full phosphorylation.

Table 1. Comparison of the *Neighbouring-effect model* with constant and variable ATP. Model selection metrics for the *Neighbouring-effect model* with variable ATP (as used in the main text) and with constant ATP concentration. Both models have the same number of parameters ($n_\theta = 7$).

	Neighbouring-effect	Neighbouring-effect w. constant ATP
**Optimisation time (mean)**	110.0s	**60.4s**
**n_θ**	7	7
**NLLH**	**-90.0**	-87.8
**AIC**	**-166.0**	-161.6
**BIC**	**-154.9**	-150.5

2 Extended models with detailed ATP binding mechanics

Throughout this work, phosphorylation is modelled as a single reaction step in which ATP is consumed directly. While this captures the net stoichiometry of the process, it does not represent the intermediate steps of the enzymatic mechanism, which include ATP binding to

the kinase, phosphate transfer, and ADP release. To test whether explicitly modelling these intermediate steps improves the description of the data, we extend the *Neighbouring-effect model* (the best-performing model in the main text) to include explicit ATP and ADP binding kinetics. We consider two variants: a fully reversible model and an irreversible model.

2.1 Reversible ATP and ADP kinetics

In the reversible variant, the kinase P-TEFb can exist in three forms: unbound, bound to ATP, and bound to ADP. The total kinase concentration is conserved:

$$[\text{P-TEFb}]_0 = [\text{P-TEFb}] + [\text{P-TEFb:ATP}] + [\text{P-TEFb:ADP}].$$

The model includes the following reactions:

$$\text{P-TEFb} + \text{ATP} \underset{r_{\text{off}}^{\text{ATP}}}{\overset{r_{\text{on}}^{\text{ATP}}}{\rightleftharpoons}} \text{P-TEFb:ATP}$$

$$\text{CTD}_{\mathbf{i}} + \text{P-TEFb:ATP} \xrightarrow{v_{\mathbf{i}\to\mathbf{j}}} \text{CTD}_{\mathbf{j}} + \text{P-TEFb:ADP}$$

$$\text{P-TEFb} + \text{ADP} \underset{r_{\text{off}}^{\text{ADP}}}{\overset{r_{\text{on}}^{\text{ADP}}}{\rightleftharpoons}} \text{P-TEFb:ADP},$$

where the phosphorylation rates $v_{\mathbf{i}\to\mathbf{j}}$ follow the same neighbouring-effect structure as in the main text, but with the kinase:ATP complex as the active species. The model introduces four additional parameters ($r_{\text{on}}^{\text{ATP}}$, $r_{\text{off}}^{\text{ATP}}$, $r_{\text{on}}^{\text{ADP}}$, $r_{\text{off}}^{\text{ADP}}$) for a total of $n_\theta = 11$. Note that we do not explicitly model the binding of the kinase complex to, and its release from, the CTD substrate. Including these steps would double the number of state variables, as each phosphorylation pattern would require both a free and a kinase-bound variant.

2.2 Irreversible ATP and ADP kinetics

In the irreversible variant, we remove the ADP rebinding reaction and the ATP unbinding reaction, assuming that once ADP dissociates from the kinase it does not rebind and that once ATP binds to the kinase it does not dissociate. This reduces the model to:

$$\text{P-TEFb} + \text{ATP} \xrightarrow{r_{\text{on}}^{\text{ATP}}} \text{P-TEFb:ATP}$$

$$\text{CTD}_{\mathbf{i}} + \text{P-TEFb:ATP} \xrightarrow{v_{\mathbf{i}\to\mathbf{j}}} \text{CTD}_{\mathbf{j}} + \text{P-TEFb:ADP}$$

$$\text{P-TEFb:ADP} \xrightarrow{r_{\text{off}}^{\text{ADP}}} \text{P-TEFb} + \text{ADP},$$

with two additional parameters ($r_{\text{on}}^{\text{ATP}}$, $r_{\text{off}}^{\text{ADP}}$) relative to the base model, for a total of $n_\theta = 9$.

Table 2. Comparison of the *Neighbouring-effect model* with different levels of ATP binding detail. Optimisation time, number of parameters (n_θ), NLLH, AIC, and BIC for the reversible, irreversible, and simplified ATP kinetics variants. Bold values indicate the best performance for each metric.

	Reversible ATP & ADP kinetics	Irreversible ATP & ADP kinetics	Simplified ATP kinetics
**Optimisation time (mean)**	2411s	**761s**	**110s**
**n_θ**	11	9	**7**
**NLLH**	**-91.6**	-87.8	-90.0
**AIC**	-161.1	-157.5	**-166.0**
**BIC**	-143.7	-143.3	**-154.9**

2.3 Comparison

Parameter estimation for both extended models was performed using the same setup as for the base model, with 128 multistart optimisation runs (Table 2). The reversible model achieves a slightly lower NLLH (-91.6) than the base model (-90.0), but at substantially increased computational cost (2411s vs. 110s per optimisation run) and with four additional parameters. The irreversible model performs worse in terms of NLLH (-87.8) despite having two additional parameters. Model selection criteria favour the base model: the simplified ATP kinetics achieve the best AIC (-166.0) and BIC (-154.9) among all three variants.

Profile likelihood analysis (Figs 1, 2) reveals that the additional binding and unbinding parameters are largely non-identifiable in both extended models. In the reversible model, k_p, $r_{\text{on}}^{\text{ATP}}$, $r_{\text{off}}^{\text{ATP}}$, and $r_{\text{off}}^{\text{ADP}}$ exhibit flat profiles spanning several orders of magnitude. This is because the base phosphorylation rate and the binding kinetics can compensate each other: a higher base rate can be offset by lower availability of the P-TEFb:ATP complex and vice versa. In the irreversible model, k_p and α are well-constrained, but $r_{\text{on}}^{\text{ATP}}$ and $r_{\text{off}}^{\text{ADP}}$ are flat and extend to their upper parameter bounds. In several profiles of both models, the sharp drops at the edges of the confidence intervals coincide with other parameters reaching their bounds during the constrained optimisation, suggesting that the apparent identifiability at those boundaries may be an artifact of the parameter bounds rather than a genuine constraint from the data.

Despite these identifiability issues, the local enhancement factor α remains well-constrained in both extended models ($\alpha \approx 2.4$ in the reversible and $\alpha \approx 2.1$ in the irreversible variant), consistent with the estimate from the base model ($\alpha \approx 2.3$). The observational parameters are similarly stable across all three models.

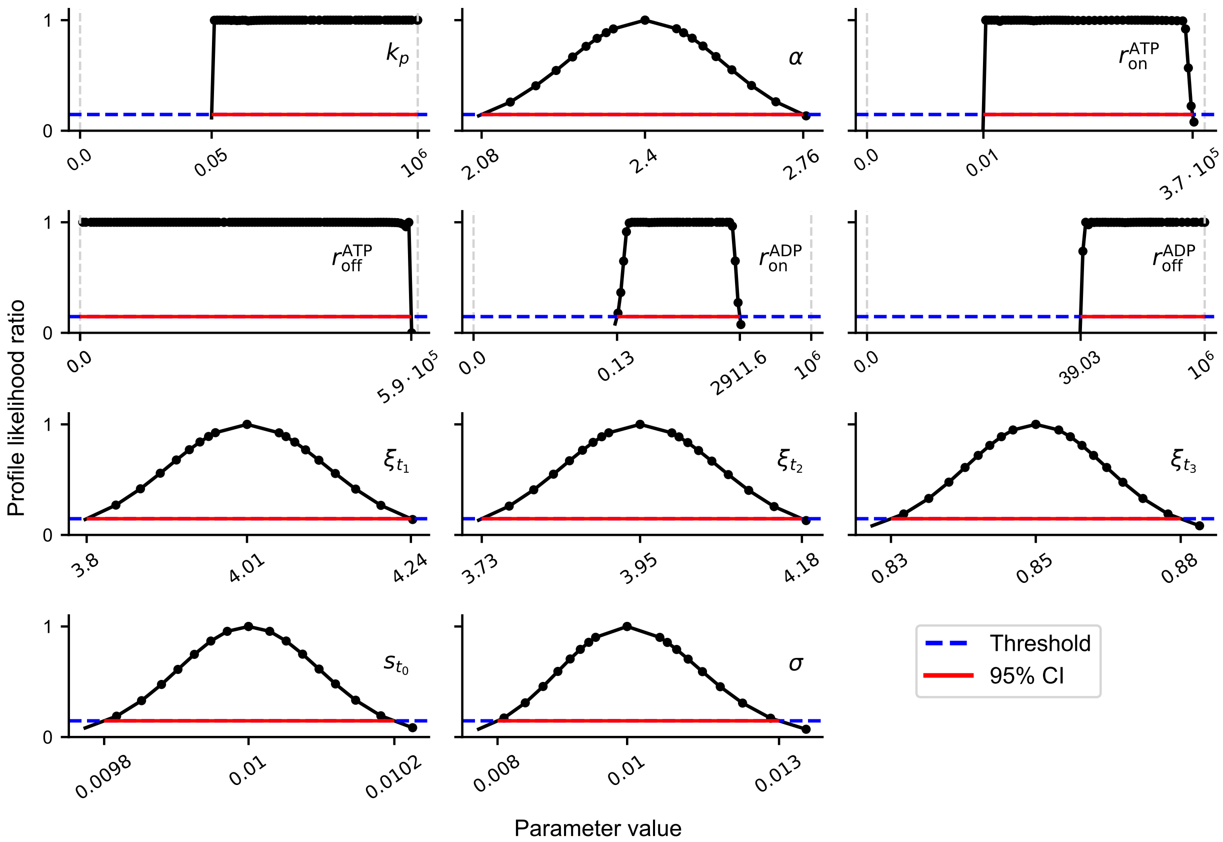

Fig 1. Profile likelihoods for the extended Neighbouring-effect model with reversible ATP and ADP kinetics. Black curves show the profile likelihood ratio for each parameter. The blue dashed line indicates the likelihood-ratio threshold, and red lines mark the 95% confidence intervals. Gray dashed lines indicate parameter bounds.

In summary, the additional mechanistic detail introduced by explicit ATP and ADP binding kinetics does not improve the model fit according to AIC and BIC, introduces substantial non-identifiabilities, and increases computational cost by more than an order of magnitude. The base *Neighbouring-effect model* with simplified ATP kinetics remains the preferred model. The robustness of α across all variants confirms that the local cooperativity finding is independent of the level of detail used to represent ATP consumption.

3 Analytical solution for the *Fully processive model*

In the main text, all models, including the *Fully processive model*, are solved numerically with ATP treated as a dynamic variable. For the *Fully processive model*, however, an analytical solution can be derived under the simplifying assumption that the ATP concentration remains constant. While this assumption has a measurable effect on the model fit under the

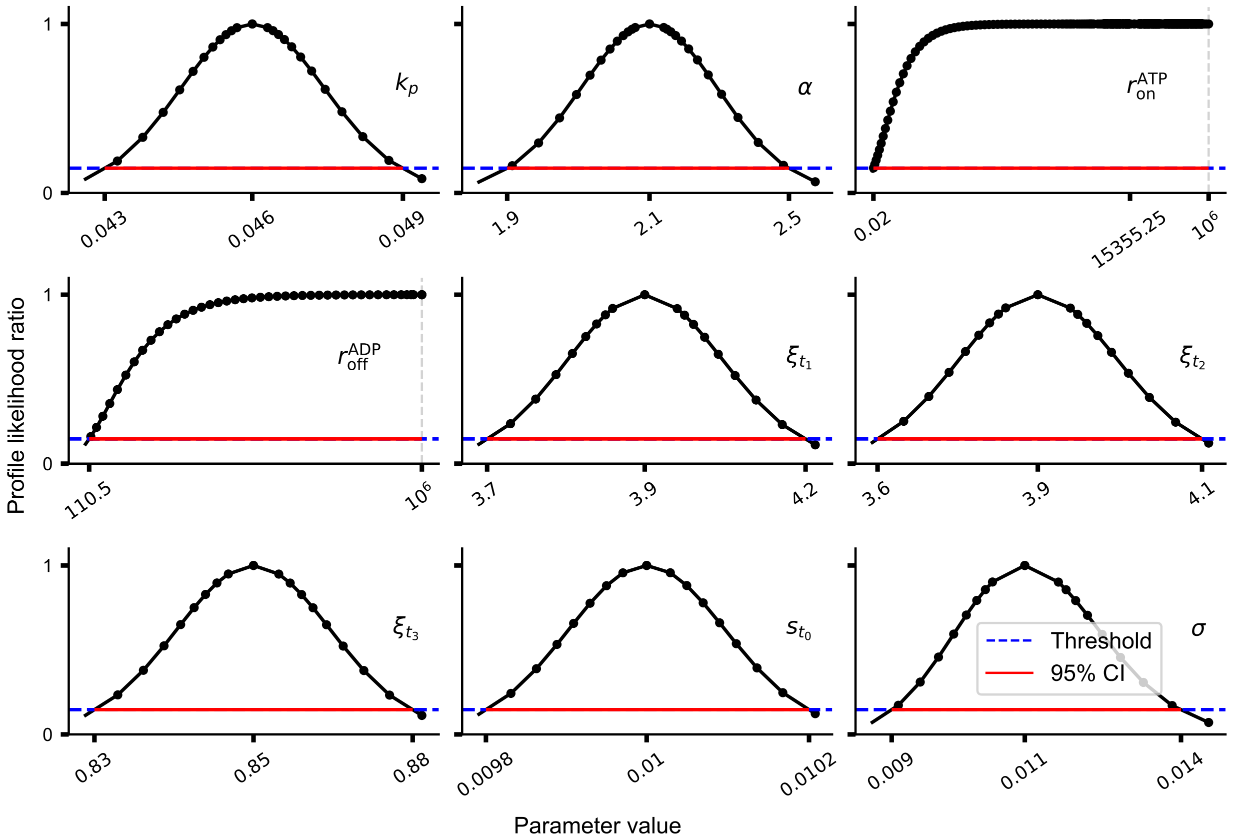

Fig 2. Profile likelihoods for the extended Neighbouring-effect model with irreversible ATP and ADP kinetics. Black curves show the profile likelihood ratio for each parameter. The blue dashed line indicates the likelihood-ratio threshold, and red lines mark the 95% confidence intervals. Gray dashed lines indicate parameter bounds.

experimental conditions considered (see Section 1), the analytical solution provides useful mathematical insight: it reveals that the processive phosphorylation dynamics follow a Poisson counting process, connecting the model to a well-known probabilistic framework. We present this derivation here as a complement to the numerical results in the main text. A similar analytical treatment is possible for the *Uniform distributive model*, without requiring the assumption of constant ATP, and is provided in the main manuscript.

The analytical solution relies on the fact that the processive phosphorylation mechanism proceeds in a consecutive manner: starting from one end of the CTD chain, a repeat can be phosphorylated only once the previous repeat has already been phosphorylated. As a consequence, only nine distinct phosphorylation patterns are possible, namely

$$\mathrm{CTD}_{[0]}, \ldots, \mathrm{CTD}_{[8]}$$

which are arranged in a simple chain of reactions. Thus, for $\ell \in \{0, \ldots, 8\}$, the total concentration of CTD chains with ℓ phosphorylated repeats is given by $[\mathrm{CTD}_{[\ell]}]$. In addition, since

the kinase is not consumed in these reactions, [P-TEFb] merely acts as a constant scalar to the reaction rate k_p. Similarly, under the assumption that ATP concentration remains constant, [ATP] also acts as a constant scalar to the reaction rate. For this reason, we define a scaled rate

$$\tilde{k}_p = [\text{P-TEFb}] \cdot [\text{ATP}] \cdot k_p,$$

which includes both scalars.

The resulting ODE system can be written in the form:

- For the unphosphorylated configuration $\text{CTD}_{[0]}$,

$$\frac{\mathrm{d}[\text{CTD}_{[0]}]}{\mathrm{d}t} = -\tilde{k}_p[\text{CTD}_{[0]}]$$

- For the intermediate configurations $\text{CTD}_{[\ell]}$ with $1 \leq \ell \leq 7$,

$$\frac{\mathrm{d}[\text{CTD}_{[\ell]}]}{\mathrm{d}t} = \tilde{k}_p([\text{CTD}_{[\ell-1]}] - [\text{CTD}_{[\ell]}])$$

- For the fully phosphorylated configuration $\text{CTD}_{[8]}$,

$$\frac{\mathrm{d}[\text{CTD}_{[8]}]}{\mathrm{d}t} = \tilde{k}_p[\text{CTD}_{[7]}].$$

As can be seen in the data, at the initial time point all CTD chains are completely unphosphorylated, i.e.,

$$[\text{CTD}_{[0]}](0) = S \quad \text{and} \quad [\text{CTD}_{[\ell]}](0) = 0 \text{ for } 1 \leq \ell \leq 8,$$

where S denotes the total substrate concentration. The cascadian structure of these ODEs allows us to calculate the solutions inductively: we begin with the completely unphosphorylated configuration whose ODE represents a simple exponential decay. Thus, its solution is given by:

$$[\text{CTD}_{[0]}](t) = Se^{-\tilde{k}_p t}.$$

Next, we assume that for an $\ell \in \{1, \ldots, 7\}$ the following has already been shown for the intermediate configuration $\text{CTD}_{[\ell-1]}$:

$$[\text{CTD}_{[\ell-1]}] = S\frac{(\tilde{k}_p t)^{\ell-1}}{(\ell-1)!}e^{-\tilde{k}_p t}.$$

The equation for $[\text{CTD}_{[\ell]}]$ takes the form

$$\frac{\mathrm{d}[\text{CTD}_{[\ell]}]}{\mathrm{d}t} + \tilde{k}_p[\text{CTD}_{[\ell]}] = \tilde{k}_p[\text{CTD}_{[\ell-1]}].$$

Applying the induction hypothesis and multiplying by $e^{\tilde{k}_p t}$ yields

$$e^{\tilde{k}_p t}\frac{\mathrm{d}[\mathrm{CTD}_{[\ell]}]}{\mathrm{d}t} + \tilde{k}_p e^{\tilde{k}_p t}[\mathrm{CTD}_{[\ell]}] = \tilde{k}_p S\frac{(\tilde{k}_p t)^{\ell-1}}{(\ell-1)!}.$$

By the reversed product rule this is equivalent to

$$\frac{\mathrm{d}}{\mathrm{d}t}\left(e^{\tilde{k}_p t}[\mathrm{CTD}_{[\ell]}]\right) = \tilde{k}_p S\frac{(\tilde{k}_p t)^{\ell-1}}{(\ell-1)!}$$

and hence

$$e^{\tilde{k}_p t}[\mathrm{CTD}_{[\ell]}] = \int_0^t \tilde{k}_p S\frac{(\tilde{k}_p s)^{\ell-1}}{(\ell-1)!}ds = S\frac{(\tilde{k}_p t)^{\ell}}{\ell!}.$$

Thus, each intermediate observable ($1 \leq \ell \leq 7$) takes the form of a scaled Poisson distribution term

$$[\mathrm{CTD}_{[\ell]}] = S\frac{(\tilde{k}_p t)^{\ell}}{\ell!}e^{-\tilde{k}_p t}.$$

For the fully phosphorylated configuration, we do not need to solve its differential equation explicitly. Instead, we can make use of the conservation of substrate. This is possible since the total amount of substrate in the system remains the same as every CTD chain must be in exactly one phosphorylation configuration and there is no degradation or synthesis taking place. Thus,

$$[\mathrm{CTD}_{[8]}] = S - \sum_{\ell=0}^{7}[\mathrm{CTD}_{[\ell]}].$$

Together, the observable mapping can be calculated as

$$y_\ell(t_k,\theta) = \begin{cases} s_{t_k}\cdot S\frac{(\tilde{k}_p t_k)^{\ell}}{\ell!}e^{-\tilde{k}_p t_k} & \text{for } 0 \leq \ell \leq 7 \\ s_{t_k}\cdot S\left(1 - \sum\limits_{\gamma=0}^{7}\frac{(\tilde{k}_p t_k)^{\gamma}}{\gamma!}e^{-\tilde{k}_p t_k}\right) & \text{for } \ell = 8. \end{cases}$$

The appearance of Poisson terms in the intermediate configurations is not accidental. In fact, the phosphorylation mechanism in the *Fully processive model* can be understood directly as a Poisson counting process: At each intermediate step, only a single phosphorylation event is possible, and each occurs with the same constant rate $\tilde{k}_p$. This setup mirrors precisely the definition of a Poisson process where events happen one at a time, independently and with a fixed rate. Let $N(t)$ be a Poisson counting process with rate $\tilde{k}_p$. Then the probability that exactly ℓ events have occurred by time t is

$$\mathbb{P}(N(t)=\ell) = \frac{(\tilde{k}_p t)^{\ell}}{\ell!}e^{-\tilde{k}_p t}.$$

Compared with our solutions, we see that the observables are simply scaled versions of these probabilities,

$$[\mathrm{CTD}_{[\ell]}] = S\cdot\mathbb{P}(N(t)=\ell).$$

However, this Poisson interpretation only holds when the concentration of ATP is approximately constant. Once ATP dynamically changes, the effective reaction rate $\tilde{k}_p(t) = k_p \cdot$ [P-TEFb] $\cdot$ [ATP](t) is no longer constant because it depends on the varying ATP concentration. Since a defining property of a Poisson process is that events occur independently and with a constant rate, this time dependence violates the Poisson assumption. Consequently, when ATP dynamics are included, the simple analytical solution derived above no longer applies, and the system must be solved numerically.
Similarly to the *Uniform distributive model*, this analytical formulation results in a substantial reduction in computational time, with each optimisation start taking on average around 0.3 seconds.

4 Global optimisation with SaCeSS

To verify that the gradient-based multi-start optimisation used in the main text identifies the global optimum, we additionally performed optimisation using SaCeSS[3], a gradient-free global optimisation method. SaCeSS is a shared-memory implementation of the Self-Adaptive Cooperative Enhanced Scatter Search algorithm, in which multiple parallel workers run enhanced scatter searches and periodically exchange promising parameter vectors. We ran SaCeSS without a local optimiser, ensuring a fully gradient-free approach.

For all three main models (*Fully processive*, *Uniform distributive*, and *Neighbouring-effect*), SaCeSS converged to the same NLLH values as the Fides-based multi-start optimisation within approximately 2 minutes of optimisation time (Fig 3). This confirms that the optima reported in the main text are not local minima and that the objective function landscape is well-behaved for these models.

5 Global-effect model

In multisite phosphorylation systems, the phosphorylation state of a substrate can globally affect the accessibility of remaining unmodified sites, for example through conformational changes or altered membrane association.[4] Such a mechanism has been discussed in the context of T cell receptor ζ-chain phosphorylation, where the unphosphorylated cytoplasmic domain associates with the plasma membrane in a conformation that is inaccessible to kinases, and phosphorylation prevents this association.[1]

To test whether an analogous global enhancement mechanism could explain the observed CTD phosphorylation dynamics, we formulate a ***Global-effect model***. This model extends the *Uniform distributive model* by scaling the phosphorylation rate once one or more sites

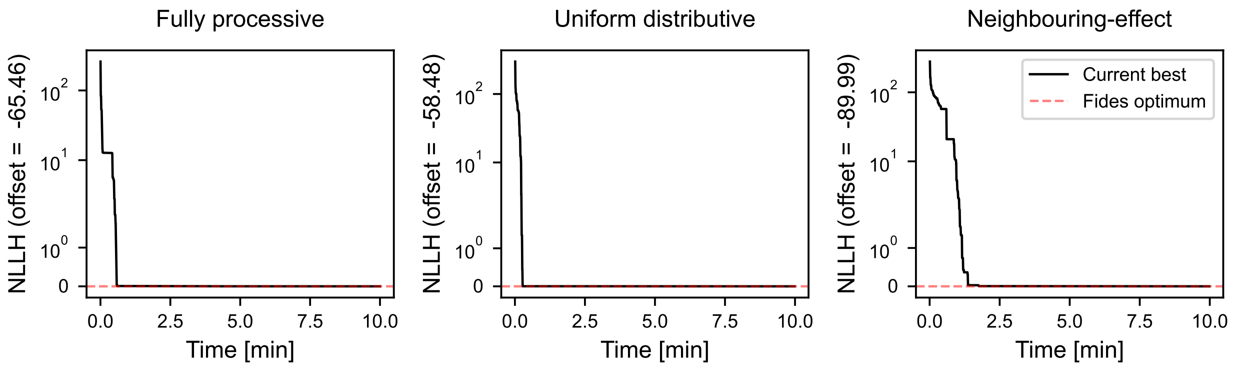

Fig 3. Convergence of the SaCeSS optimiser for the three main models. The best NLLH found is shown as a function of optimisation time for the *Fully processive* (left),*Uniform distributive* (centre), and *Neighbouring-effect* (right) models. Dashed orange horizontal lines indicate the optimal NLLH obtained by Fides multi-start optimisation in the main text. SaCeSS converges to the same values for all models.

are already phosphorylated. Using the same notation as in the main manuscript, the rates are defined as

$$v_{i \to j} = \begin{cases} k_p \cdot \alpha, & \text{if } \|i\|_1 \geq 1 \\ k_p, & \text{otherwise.} \end{cases}$$

The model thus has the same number of parameters ($n_\theta = 7$) as the *Neighbouring-effect model*: a base phosphorylation rate k_p, a global enhancement factor α, and five observational parameters.

The waterfall plot indicates good convergence of the optimisation (Fig 4A). The *Global-effect model* achieves an NLLH of -72.8, which is substantially worse than the *Neighbouring-effect model* (-90.0) despite having the same number of parameters (Fig 4B). This is also reflected in the AIC (-131.5 vs. -166.0) and BIC (-120.4 vs. -154.9). A comparison of the best-fit simulations (Section 6, Fig 5) shows that the *Global-effect model* underestimates the 1P peak at $t = 1$ h and overshoots intermediate states, similar to the *Uniform distributive model.* This is consistent with the model structure: after the first phosphorylation event, all remaining sites are enhanced equally, so the model cannot produce the graded, position-dependent rate differences that the data require.

6 Comparison of model fits

To complement the quantitative model selection presented in the main text, we compare the best-fit time-course simulations of all model variants against the experimental data (Fig 5). This comparison allows a qualitative assessment of where each model succeeds or fails in

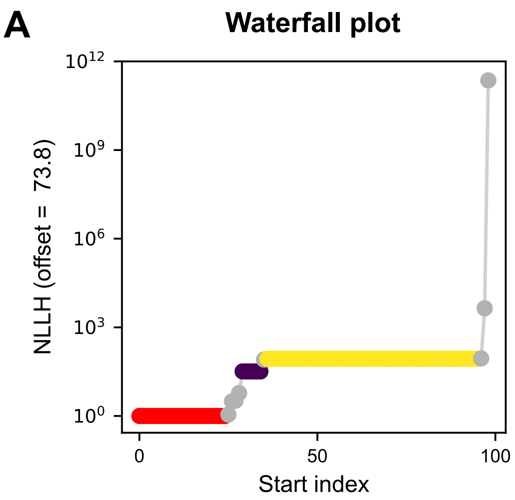

B Optimisation results

	Global-effect
**Optimisation time (mean)**	128.1s
$\mathbf{n_\theta}$	7
**NLLH**	-72.8
**AIC**	-131.5
**BIC**	-120.4

Fig 4. Waterfall plot and optimisation results for the *Global-effect model.* (A) Waterfall plot of sorted NLLH values across optimisation starts. (B) Optimisation results including mean computation time, number of parameters (n_θ), NLLH, AIC, and BIC.

reproducing the observed phosphorylation dynamics.

All four models capture the general trend of the data: depletion of the unphosphorylated state (0P), transient accumulation of intermediate phosphorylation states, and eventual progression towards full phosphorylation (8P) at 16 h. However, the models differ in how accurately they reproduce the shape and timing of these transitions.

The *Uniform distributive model* shows the largest deviations from the data. It depletes the 0P state too rapidly at early time points and underestimates the 1P peak at $t = 1$ h. It also overestimates 3P at $t = 1$ h and 4P at $t = 2$ h. These deviations reflect the model's single-rate assumption: with a uniform phosphorylation rate k_p for all sites regardless of context, the model cannot independently capture both the slow initial depletion of 0P and the accelerated progression through intermediate states observed in the data.

The *Global-effect model* (see Section 5 for a full description) shows a pattern of deviations similar to the *Uniform distributive model*, particularly in the underestimation of the 1P peak at $t = 1$ h and the overshoot of intermediate states such as 4P. The global-effect model applies a uniform rate enhancement to all sites once any single site is phosphorylated. This introduces an additional degree of freedom relative to the *Uniform distributive model*, allowing the model to better capture the initial depletion of 0P by distinguishing the rate of the first phosphorylation event from all subsequent ones. However, after the first phosphorylation, all remaining sites are enhanced equally, so the model shares the qualitative shortcomings of the *Uniform distributive model* for intermediate and late phosphorylation states.

The *Fully processive model* provides a better fit to the data across most phosphorylation

states, outperforming the *Uniform distributive model* despite also having a single rate parameter. The most notable discrepancy in the fit is at the highly phosphorylated states (7P and 8P), where the model predicts faster accumulation than is observed at $t = 2$ h. As with the other single-rate models, the model cannot independently adjust the dynamics at different stages of phosphorylation.

The *Neighbouring-effect model* provides the closest agreement with the data across all phosphorylation states and time points. Unlike the uniform and global-effect models, the rate enhancement in this model is conditional on the local phosphorylation configuration, which produces a distribution over intermediate states that more closely matches the observed dynamics. Unlike the processive model, it does not impose a fixed sequential order, allowing for more flexible progression patterns that better capture the timing of late phosphorylation events.

These qualitative observations are consistent with the quantitative model selection results presented in the main text, where the *Neighbouring-effect model* achieves substantially lower NLLH, AIC, and BIC values (ΔAIC, ΔBIC > 10 relative to all other models).

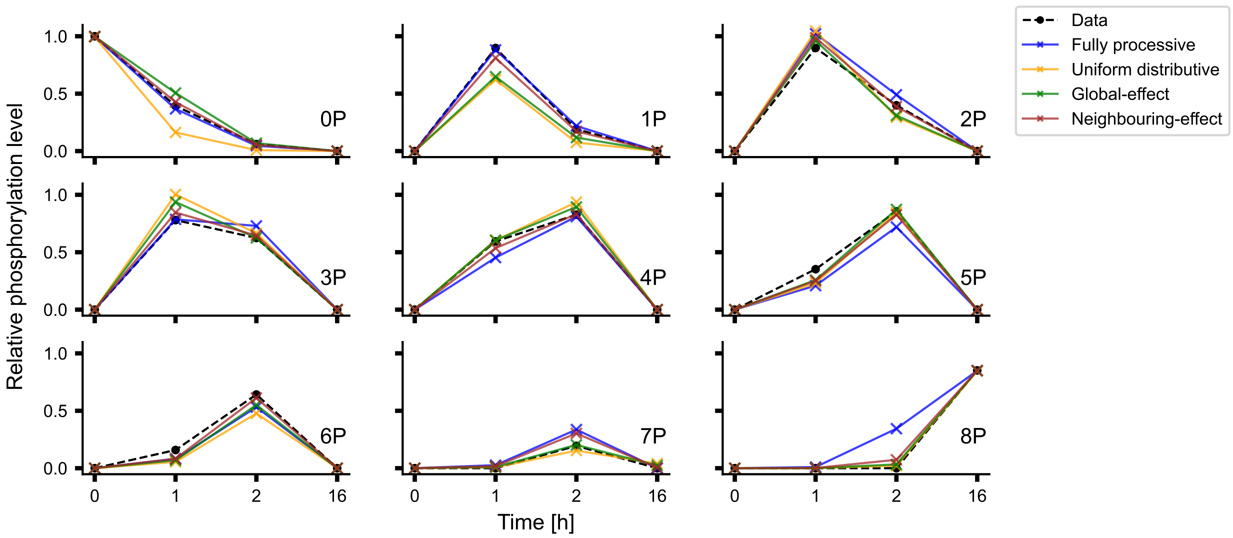

Fig 5. Comparison of best-fit simulations across model variants. Best-fit time-course simulations for the *Fully processive model* (blue), the *Uniform distributive model* (orange), the *Global-effect model* (green), and the *Neighbouring-effect model* (red) are shown alongside the experimental data (black dashed). Each panel corresponds to a phosphorylation count (0P–8P). The *Neighbouring-effect model* provides the closest agreement with the data across all states.

References

[1] D. Aivazian and L. J. Stern. Phosphorylation of T cell receptor ζ is regulated by a lipid dependent folding transition. *Nature Structural Biology*, 7(11):1023–1026, Nov. 2000. ISSN 1545-9985.

[2] N. Czudnochowski, C. A. Bösken, and M. Geyer. Serine-7 but not serine-5 phosphorylation primes RNA polymerase II CTD for P-TEFb recognition. *Nature Communications*, 3(1):842, May 2012. ISSN 2041-1723.

[3] D. R. Penas, P. González, J. A. Egea, R. Doallo, and J. R. Banga. Parameter estimation in large-scale systems biology models: A parallel and self-adaptive cooperative strategy. *BMC Bioinformatics*, 18(1):52, Jan. 2017. ISSN 1471-2105.

[4] C. Salazar and T. Höfer. Multisite protein phosphorylation – from molecular mechanisms to kinetic models. *The FEBS Journal*, 276(12):3177–3198, 2009.
