# Supplementary figures and images for: Quantitative modelling of P-TEFb mediated CTD phosphorylation identifies local cooperativity
Source: PLoS Comput Biol. 2026 Jul 30;22(7):e1014531. doi: 10.1371/journal.pcbi.1014531 (PMC13423041; doi:10.1371/journal.pcbi.1014531)

Likelihood ratio

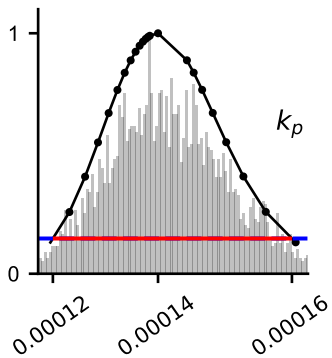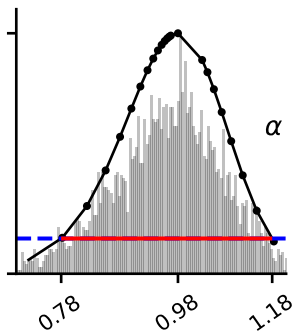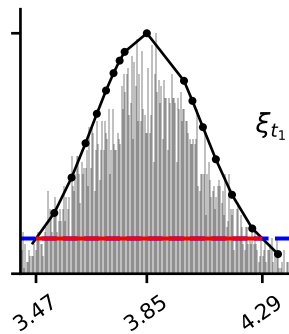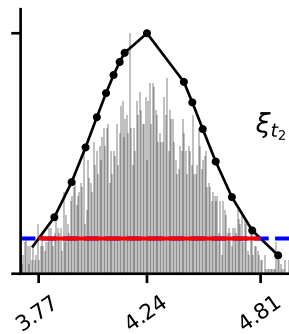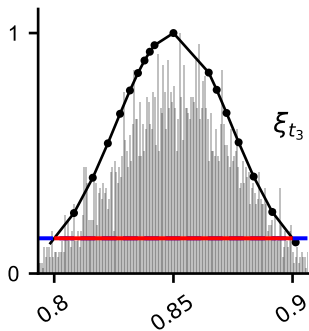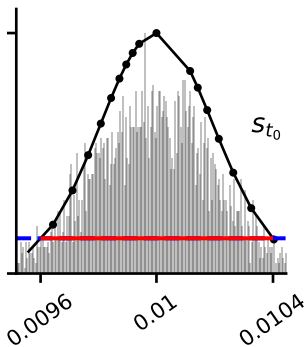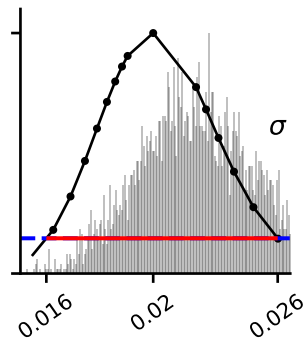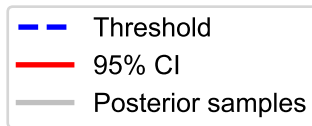

Geweke burn-in index: 20000

Parameter value

Supplement: S1 Fig — Black curves show the obtained profile likelihood (maximum likelihood obtained for the fixed parameter value) normalised by the maximum likelihood from optimisation. The red line marks the 95% confidence threshold, and the blue dashed line indicates the CI cutoff. Gray histograms show the marginal posterior distributions obtained via MCMC sampling. (PDF) [file pcbi.1014531.s002.pdf]

Likelihood ratio

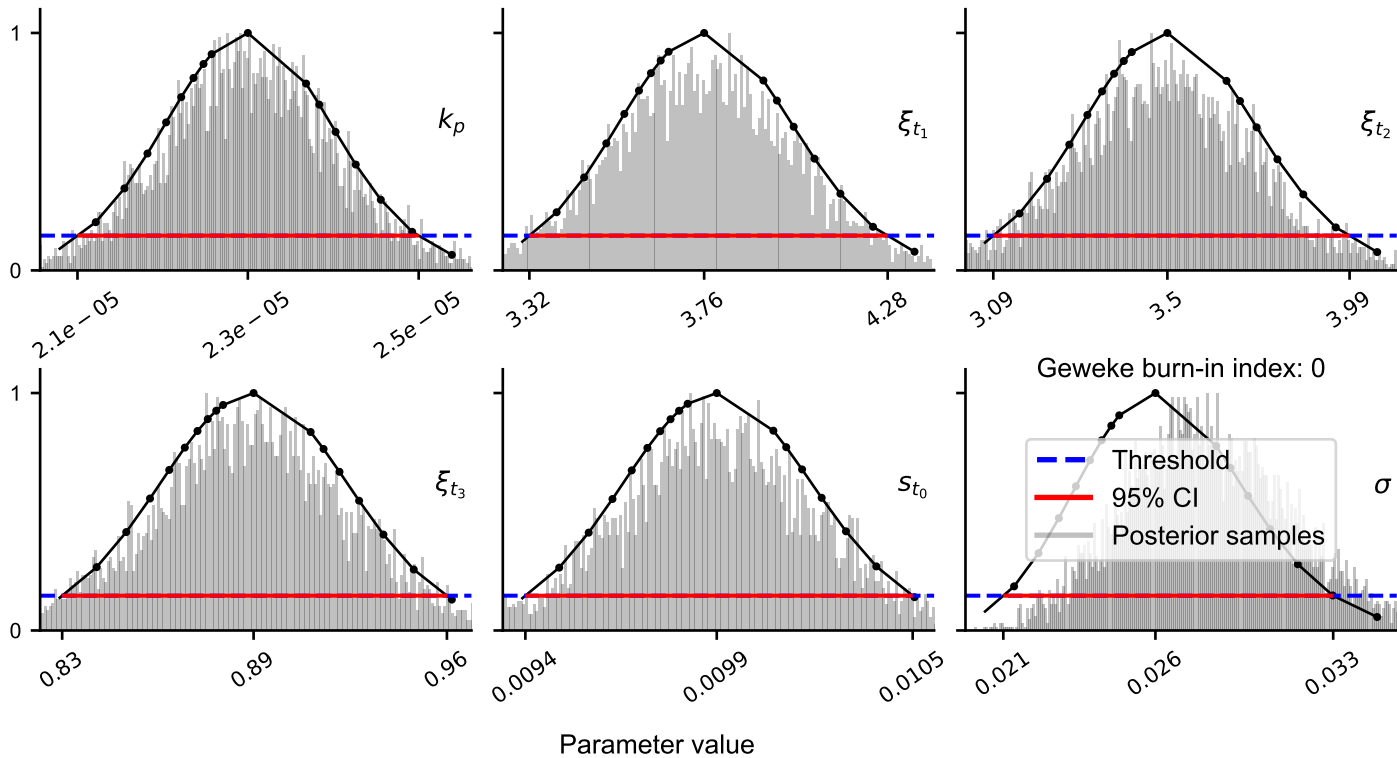

Supplement: S2 Fig — Black curves show the obtained profile likelihood (maximum likelihood obtained for the fixed parameter value) normalised by the maximum likelihood from optimisation. The red line marks the 95% confidence threshold, and the blue dashed line indicates the CI cutoff. Gray histograms show the marginal posterior distributions obtained via MCMC sampling. (PDF) [file pcbi.1014531.s003.pdf]

Likelihood ratio

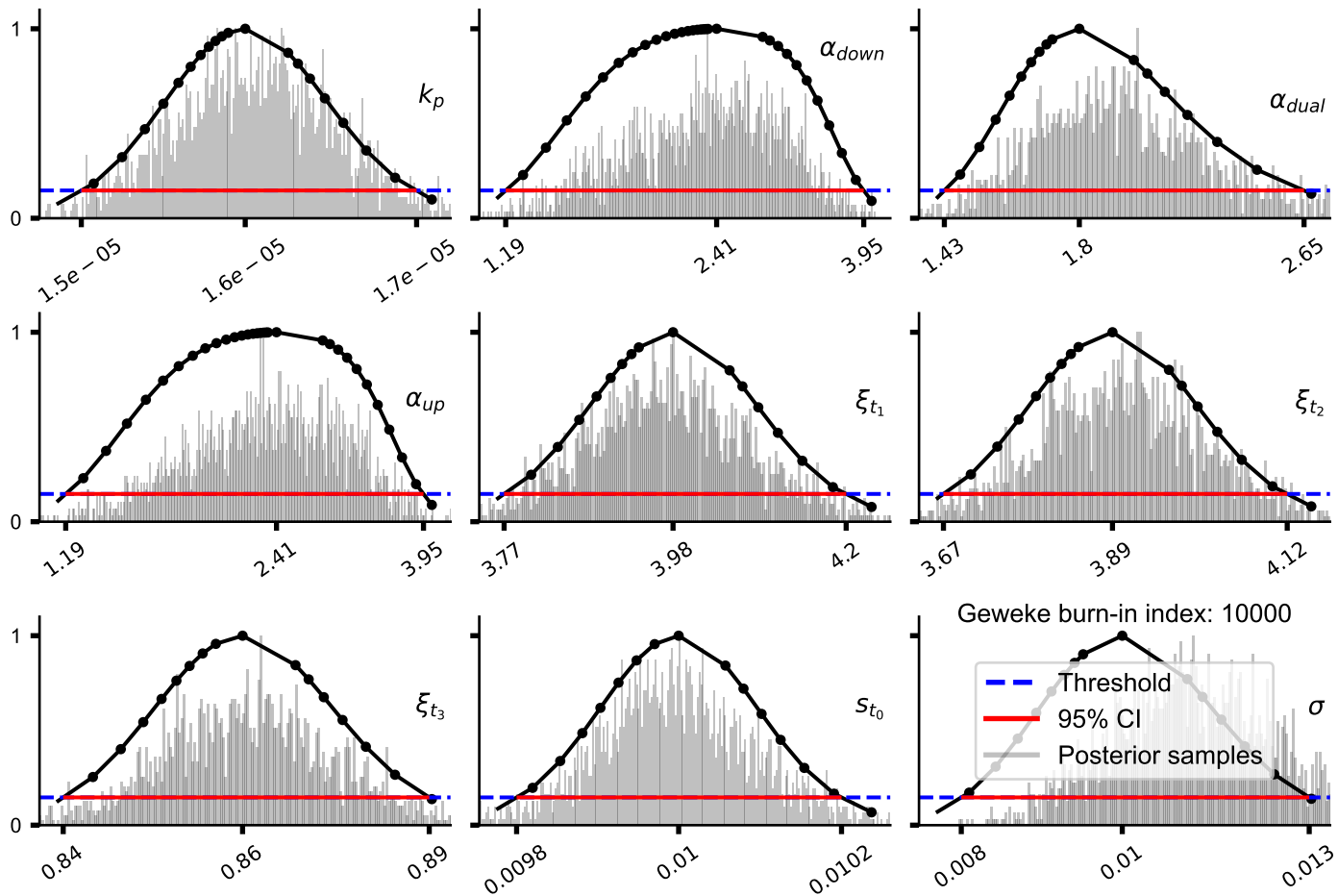

Parameter value

Supplement: S3 Fig — Black curves show the obtained profile likelihood (maximum likelihood obtained for the fixed parameter value) normalised by the maximum likelihood from optimisation. The red line marks the 95% confidence threshold, and the blue dashed line indicates the CI cutoff. Gray histograms show the marginal posterior distributions obtained via MCMC sampling. (PDF) [file pcbi.1014531.s004.pdf]
